# Non-Invasive, Topical Sampling of Potential, Low-Molecular Weight, Skin Cancer Biomarkers: A Study on Healthy Volunteers

**DOI:** 10.1021/acs.analchem.1c05470

**Published:** 2022-04-08

**Authors:** Skaidre Jankovskaja, Maxim Morin, Anna Gustafsson, Chris D. Anderson, Boglarka Lehoczki, Johan Engblom, Sebastian Björklund, Melinda Rezeli, György Marko-Varga, Tautgirdas Ruzgas

**Affiliations:** † Department of Biomedical Science, Malmö University, Malmö 214 28, Sweden; ‡ Biofilms—Research Center for Biointerfaces, Malmö University, Malmö 214 28, Sweden; § Department of Biomedical and Clinical Sciences, Linköping University, Linköping 581 83, Sweden; ∥ Department of Dermatology and Venereology, Linköping 581 83, Sweden; ⊥ Clinical Protein Science and Imaging, Department of Biomedical Engineering, Lund University, Lund 221 00, Sweden

## Abstract

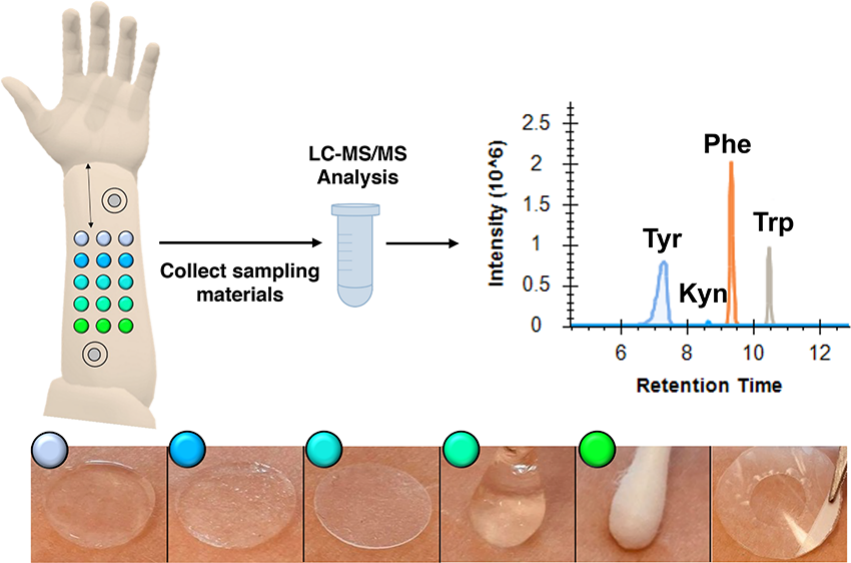

Monitoring of low-molecular weight cancer biomarkers, such as tryptophan (Trp) and its derivative kynurenine (Kyn), might be advantageous to non-invasive skin cancer detection. Thus, we assessed several approaches of topical sampling of Trp and Kyn, in relation to phenylalanine (Phe) and tyrosine (Tyr), on the volar forearm of six healthy volunteers. The sampling was performed with three hydrogels (made of agarose or/and chitosan), hydrated starch films, cotton swabs, and tape stripping. The biomarkers were successfully sampled by all approaches, but the amount of collected Kyn was low, 20 ± 10 pmol/cm^2^. Kyn quantification was below LOQ, and thus, it was detected only in 20% of topical samples. To mitigate variability problems of absolute amounts of sampled amino acids, Tyr/Trp, Phe/Trp, and Phe/Tyr ratios were assessed, proving reduced inter-individual variation from 79 to 45% and intra-individual variation from 42 to 21%. Strong positive correlation was found between Phe and Trp, pointing to the Phe/Trp ratio (being in the 1.0–2.0 range, at 95% confidence) being least dependent on sampling materials, approaches, and sweating. This study leads to conclusion that due to the difficulty in quantifying less abundant Kyn, and thus the Trp/Kyn ratio, the Phe/Trp ratio might be a possible, alternative biomarker for detecting skin cancers.

## Introduction

Skin cancer is curable in most patients if detected before the establishment of a metastatic phenotype, which underlines the importance of early diagnosis.^1^ To date, visual inspection followed by biopsy is the gold standard of skin cancer diagnosis.^1,2^ However, at an early stage, the visual diagnostic approach has limited specificity (<30%) and sensitivity (approx. 84%).^2^ This means that out of 100 melanoma-judged cases, and thus decided for excision, only 30 are melanomas. An 84% sensitivity implies that 16% out of the true melanoma cases will be misdiagnosed. Therefore, additional simple non-invasive tools to support skin cancer diagnosis are highly desired by both health care providers and patients.^2^

Melanoma develops at the basal membrane of the epidermis, where abnormal proliferation of melanocytes starts.^3^ The cancerous cells then grow and reach upper skin layers, blood vessels, and the lymphatic system in the dermis, facilitating metastasis.^4,5^ The upward-growing melanoma cells, which at the end get shed on the epidermal surface,^4^ expose two-four week old high-molecular weight (HMW) tumor biomarkers. This delay is determined from the kinetics of terminal differentiation of the keratinocytes, leading to shedding of corneocytes.^6^ Contrarily, the tumor microenvironment (TME) could be much quicker exposed on the skin surface with low-molecular weight (LMW) cancer biomarkers. It is well known that due to barrier properties of the stratum corneum (SC), permeation of HMW substances through skin is strongly restricted, while LMW (<500 Da) compounds can permeate the SC and reach the surface of the skin in hours.^7^

Non-invasive, topical collection of LMW analytes was previously carried out by employing various sampling techniques, which are summarized in several review articles.^8,9^ For instance, hydrophilic LMW analytes were successfully collected from the human skin surface using agarose hydrogel^10^ or commercially available peelable gel^11^ or by exposing skin to phosphate-buffered saline (PBS)^12^ and were analyzed by desorption electrospray ionization mass spectrometry (DESI–MS), liquid chromatography mass spectrometry (LC–MS), or nuclear magnetic resonance (NMR), respectively. Moreover, non-invasive skin metabolite collection with a hydrogel micropatch demonstrated the feasibility to detect statistically significant differences between psoriatic and healthy skin.^13,14^ By measuring the abundance of citrulline and choline on the skin surface, the research group could follow the treatment of skin psoriasis.^14^ Interestingly, the temporal changes of these metabolites were detected on the skin surface during psoriatic skin treatment, while these changes were not reflected in blood.^14^ All these results suggest that non-invasive LMW biomarker monitoring might capture the dynamics of metabolic changes in the TME and be useful in improving skin cancer diagnostics.

To the best of our knowledge, LMW biomarkers have not yet been assessed for non-invasive skin cancer diagnostics. Therefore, we investigated the possibility to collect tryptophan (Trp) and its metabolite kynurenine (Kyn) and the ratio of Trp to Kyn (Trp/Kyn) on the surface of skin of healthy volunteers. Trp plays an important role in health and disease via its involvement in three major metabolic pathways: production of serotonin, protein synthesis, and the Kyn pathway (KP).^15^ In the KP, Trp is converted into biologically active metabolites, including Kyn, by three rate-limiting enzymes, that is, indoleamine 2,3-dioxygenase 1 and 2 (IDO-1 and IDO-2) and Trp 2,3-dioxygenase.^15^ IDO-1 expression was reported to be upregulated in numerous malignancies such as lung cancer,^16^ renal cell carcinoma,^17^ melanoma,^17−20^ and others.^18^ The upregulated expression of IDO-1 leads to Trp depletion and Kyn generation, resulting in a decrease in the Trp/Kyn ratio in the TME^21^ and the melanoma patient’s plasma,^17,22^ which correlates with the patient’s survival expectation. Because both Trp and Kyn are LMW compounds and are able to diffuse across the skin barrier,^23^ we expect that concentrations of Trp and Kyn, and possibly the Trp/Kyn ratio, present in the TME can be reproduced on the skin surface. To consider testing this hypothesis in clinics, a highly reproducible methodology for Trp and Kyn sampling from the skin surface is needed. In order to achieve this goal, it is very important to oversee possible factors causing variability of these biomarkers, for example, by sweating, sampling procedure, or analysis protocols. To build confidence in analytical procedures, we also investigated non-invasive sampling of Trp and Kyn, in relation to other amino acids, phenylalanine (Phe) and tyrosine (Tyr). The sampling of two additional amino acids is motivated by the fact that they, like Trp, constitute the natural moisturizing factor (NMF) pool in the SC.^24^ Therefore, assessing Phe and Tyr in relation to Trp can provide an extra control for discovering and minimizing Trp and Kyn sampling errors.

Keeping in mind the motivation discussed above, the overall aim of this study was to assess a few strategies for non-invasive sampling of cancer-related biomarkers, Trp and Kyn in relation to Tyr and Phe, under two clinically relevant conditions: at rest and while sweating. The study included only healthy volunteers. The sampling of the biomarkers was carried out using hydrogels, a potato starch film, cotton swabs, and tape stripping techniques (Table 1). The influence of the sampling approaches on the skin barrier was evaluated by electrical impedance spectroscopy (EIS). The comparison between sampling approaches was based on the quantities of collected analytes and their ratios. The quantities and the ratios were also compared to blood levels. The obtained results showed that the absolute quantities, collected by different sampling approaches and sampling materials, differ and can also be affected by sweating. However, the impact of these factors was strongly reduced by considering ratios of the analyzed amino acids.

## Experimental Section

### Study Participants

Six healthy Caucasian volunteers (three men and three women in the age range 25–35 years) with no history of previous or ongoing skin disease on the volar side of the arm were included in this study. The study was approved by the Swedish Ethical Review Agency (Dnr 2021-03784). Subjects were asked not to apply any skin care products on their forearms 24 h before the study. Participants were also asked not to use detergents on their forearms 12 h before the study. No dropouts and no side effects were observed throughout the study.

### Collection and Extraction of Biomarkers

**Figure 1:**
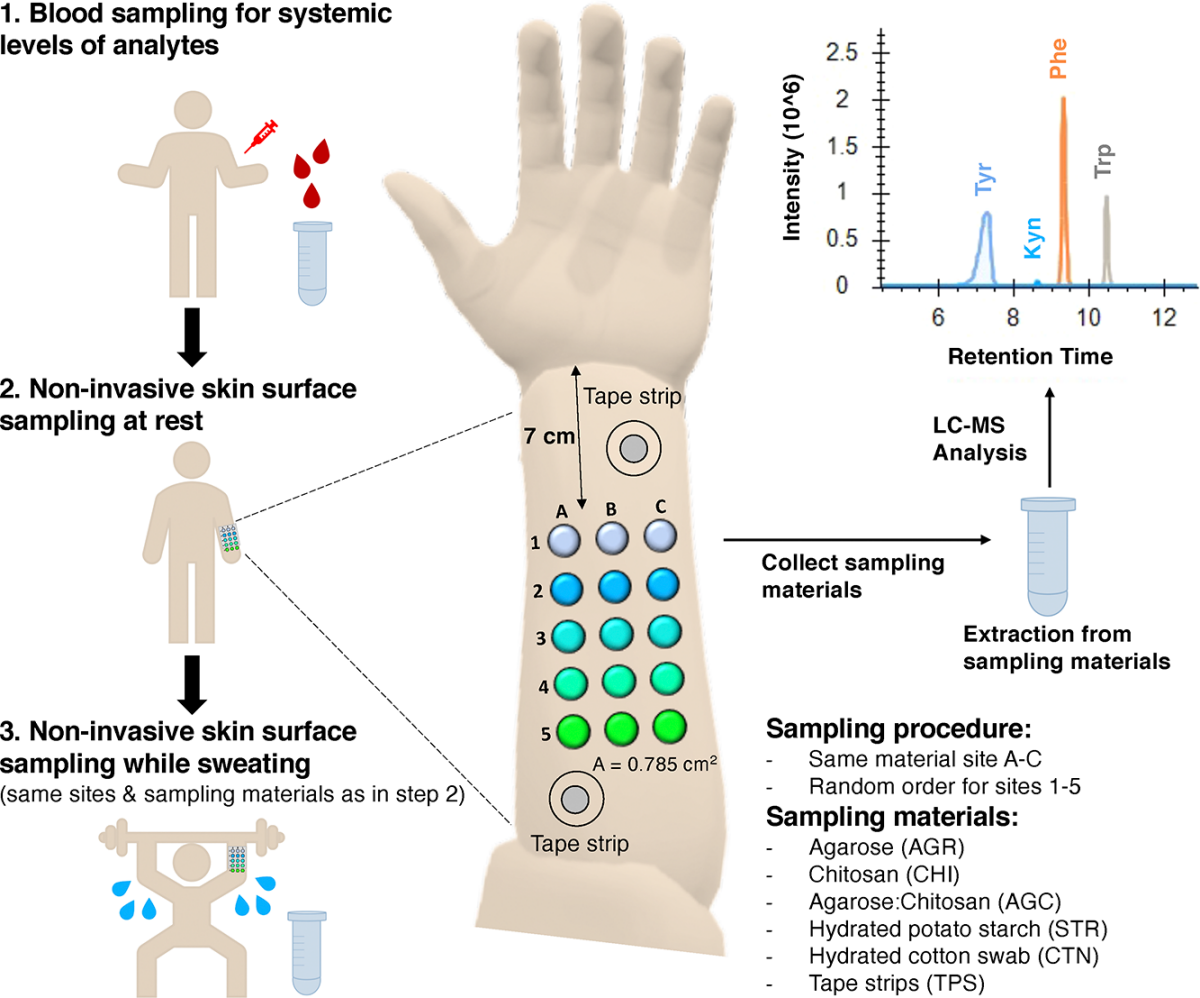
Schematics of the study design representing sampling of LMW biomarkers Trp, Kyn, Phe, and Tyr from blood plasma and from the skin surface under two conditions: at rest and sweating induced by physical activity. A, B, and C indicate placement of the same material or same procedure of sampling. 1, 2, 3, 4, and 5 indicate random placement of sampling material. Collected samples were subjected to extraction followed by LC–MS analysis.

Chemicals used for the study can be found in Section S1 (see Supporting Information). Prior the skin surface sampling, a marked skin area was cleaned with a Salvequick wound cleanser (Orkla, Solna, Sweden). Each sampling site with an area of 0.785 cm^2^ and a 2 cm distance between each site was defined by applying a custom-made frame from the Chemotechnique skin patch (Figure S1, Chemotechnique MB Diagnostics AB, Vellinge, Sweden). LMW biomarkers were collected from the test sites using six different sampling materials, summarized in Table 1. Sampling was performed on the volar forearms at 17 different sampling sites per individual, Figure 1. Sampling with the same material, except for tape sampling (TPS), was performed at three adjacent sampling sites (A, B, and C in Figure 1). Placement of sampling materials (1, 2, 3, 4, and 5 in Figure 1) was randomly varied between individuals. TPS was performed at two sampling sites; one sampling site close to the wrist and one close to the elbow. Three tape strips were collected from the same sampling site and pooled together for analysis. Sampling of biomarkers with hydrated cotton swab (CTN) was performed by wiping the skin surface for 5 s. The hydrogels (agarose (AGR), chitosan (CHI), and agarose:chitosan mixture (AGC)) and hydrated starch film (STR) were applied and kept on the skin surface for 2 h under occlusion. To assess the loss of water from these materials, they were weighted before and after the application. After sampling, all materials were stored at −80 °C. The extraction of analytes was performed by adding 1 mL of 20% (v/v) MeOH in Milli-Q water solution or, in the case of CHI and AGC, 131 mM NaCl at a pH of 12 and shaking it at 400 rpm (Heidolph Titramax 100, Buch and Holm, Herlev, Denmark) for 1 h. After extraction, the Eppendorf tubes were centrifuged (Multifuge 3 S-R, Heraeus, Germany) at 12,000 x g for 15 min at 20 °C, and the supernatant was filtered with a syringe filter (13 mm, w/0.2 μm PTFE membrane, VWR International, US). Then, the filtered supernatant was concentrated 10 times by drying in a centrifugal evaporator (EZ-2 Plus evaporating system, Genevac LTD., England) and re-suspending in 0.1 mL of 20% MeOH. Samples collected from skin surfaces during extensive sweating were treated in the same way, excluding the pre-concentration step.

**Table 1:**
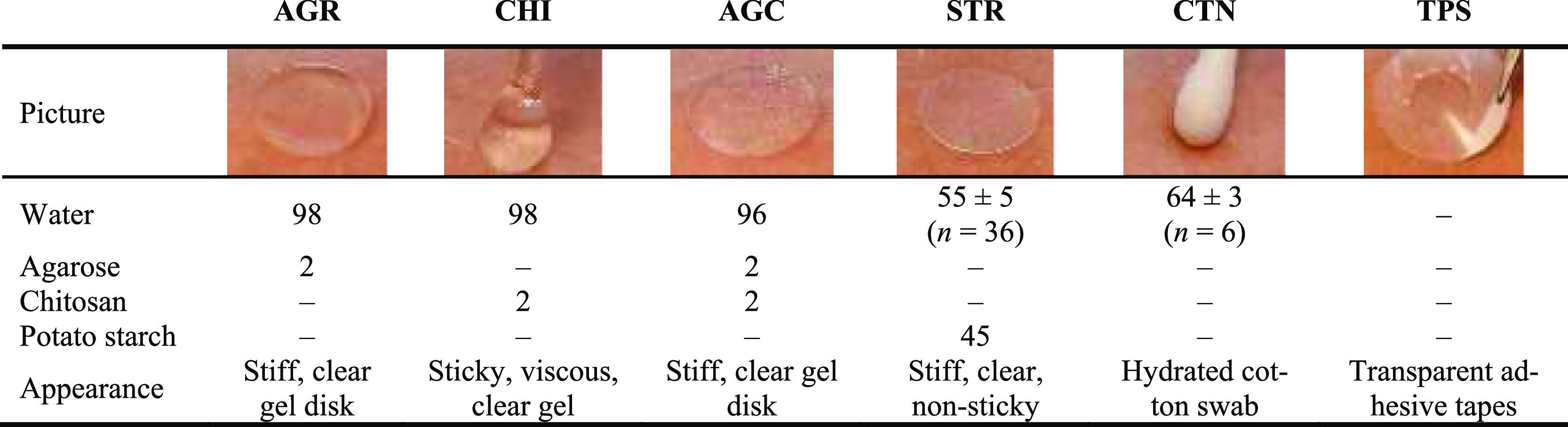
Composition of Materials Used for Non-Invasive Sampling of Biomarkers from the Skin Surfaces: Agarose (AGR), Chitosan (CHI), a Combination of Agarose and Chitosan (AGC), Hydrated Starch (STR), Hydrated Cotton Swabs (CTN), and Tape Stripping (TPS)^a^

a Data show mean ± SD where appropriate. Detailed sampling material preparation procedures are described in Section S2.

Prior to sampling of biomarkers from the skin surface, the fasting blood samples were collected, and the quantities of analytes were measured (for more details see Section S3). The physical barrier properties of the sampling sites were assessed by means of transepidermal water loss (TEWL) and EIS before and after the sampling (for detailed procedure of the measurements, see Section S4).

### LC–MS/MS Analysis

A Micromass Quattro micro-Tandem Quadrupole mass spectrometer (QAA009, Waters, Mildford, MA) equipped with an ESI ion source and coupled with an Alliance high-performance liquid chromatography system (2795 Waters, Mildford, MA) was used for quantification of Tyr, Kyn, Phe, and Trp. The analytes were separated on an analytical Kromasil C18 column (5 μm particle size, pore size 100 Å, L × I.D. 250 × 4.6 mm from ES industries, West Berlin NJ, USA). Solvents A (0.1% formic acid in water) and B (0.1% formic acid in methanol) were used to create a 25 min linear gradient to elute the analytes. The gradient profile was as follows: 10% solvent B was increased to 90% over 15 min at a flow rate of 0.5 mL/min and held at 90% B for 5 min at a flow rate of 0.8 mL/min; then solvent B was decreased to 10% in 0.1 min and kept at 10% B for 4.9 min at a flow rate of 0.5 mL/min. The LC–MS/MS measurements were carried out in the multiple reaction monitoring (MRM) mode, while operating in positive polarity. The capillary voltage was set to 3.05 kV, and the source temperature was kept at 110 °C. Desolvation gas flow was set to 900 L/h, cone gas flow was set to 25 L/h, and the desolvation temperature was raised to 400 °C. The collision gas pressure in *Q*2 was set to 8.8 × 10^–4^ Torr. The interchannel delay was 0.1 s, and interscan delay was 0.1 s. The span window was set to 1 Da. The MRM transitions and parameter values used to measure the analytes are listed in Table S2.

Data analysis was performed by using Skyline v 21.1 software (MacCoss Lab Software, Seattle, WA, USA). The unknown concentrations of analytes were calculated based on the calibration standards in the range from 0.9 to 240 pmol for Trp and Kyn, from 3.8 to 240 pmol for Phe, and from 15 to 960 pmol for Tyr (*R*^2^ > 0.99) (precision, LODs and LOQs can be found in Table S3). Description of the preparation procedure of stock solutions and calibration standards can be found in Section S5. The evaluation of the matrix effect, recoveries, and overall process efficiency for different sampling techniques are summarized in Table S4 and Figure S2.

### 3D Cell-Cultured Skin Models

The procedure to obtain the three-dimensional (3D) cell-cultured skin model is described in Section S6. The 3D cell-cultured skin consisting of a fibroblast-populated dermis and a fully differentiated epidermis was stimulated with interferon-gamma (IFN-γ) or UV-B irradiation. Appropriate amount of IFN-γ was added to the medium under the skin holding insert to obtain final concentrations of 10, 20, and 50 ng/mL of IFN-γ in EpiLife cell culture medium. After 48 h of stimulation with IFN-γ, the culture medium was collected and aliquots were immediately frozen at −80 °C. For the UV-B treatment, the culture medium was removed, cells were washed with PBS, and the 3D cultures were irradiated with UV-B (302 nm) at doses of 40 or 80 mJ/cm^2^ (UVM-57 UV lamp, Analytik Jena, USA). Then, fresh medium was added, and the cultures were maintained at the air–liquid interface (ALI) for 48 h. After 48 h, the culture medium was collected and aliquots were immediately frozen at −80 °C. The quantities of Tyr, Phe, Trp, and Kyn in cell culture aliquots were measured by LC–MS/MS.

### Statistical Analysis

All statistical analyses were performed using RStudio (Version 1. 3. 1093, PBC, Boston, MA, USA). Data are reported as mean ± SD unless otherwise stated. The distribution of the residuals of the data was checked by doing two formal normality tests, Shapiro–Wilk and Kolmogorov–Smirnov, and visually inspecting quantile–quantile plots. Homogeneity of the variances was checked by carrying out Levene’s test. Comparison of two related groups of data was carried out by using paired-sample *t*-test. In cases where the normality assumption was not fulfilled for two related group comparisons, non-parametric Wilcoxon singed-rank test was used. For three or more groups of data, one-way Anova with posthoc Tukey test was applied. Alternatively, if assumptions of homogeneity of variances were not justified, Kruskal Wallis test followed by Wilcoxon rank-sum test with false discovery rate correction was performed. Correlations between different analytes were evaluated by Spearman’s rank test. Significance levels: “*” *p* < 0.05, “**” *p* < 0.01, and “***” *p* < 0.001.

Each data set, used for statistical analysis, held data values taken at each sampling site considering them as independent observations (*n* = 17 sampling sites per individual; *n* = 3 (A–C) × 6 (volunteers) = 18 sampling sites per sampling technique, see Figure 1). Ratios between the analytes were determined for each sample separately and then averaged.

## Results and Discussion

### Quantities of Tyr, Phe, Trp, and Kyn and Their Ratios, Sampled by Different Techniques

**Figure 2:**
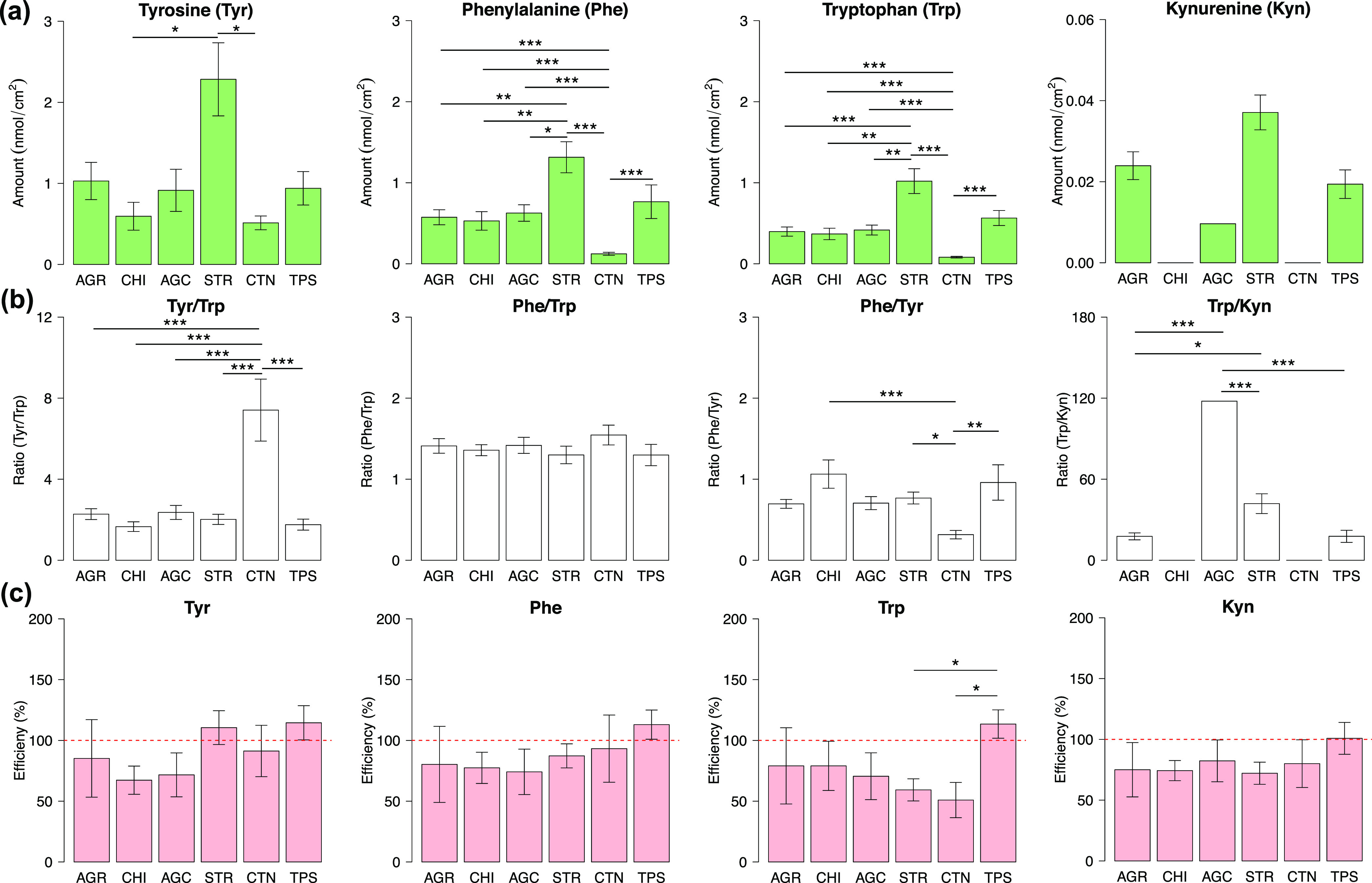
Comparison of different sampling approaches to collect biomarkers from the skin surface. The absolute amounts of Tyr, Phe, Trp, and Kyn (a) and the Tyr/Trp, Phe/Trp, Phe/Tyr, and Trp/Kyn ratios (b). Biomarkers were collected during 2 h sampling with different hydrogels (AGR, AGC, and CHI) and hydrated starch films (STR) or by “instant sampling” with a hydrated cotton swab (CTN) and by tape stripping (TPS). Data in (a,b) show mean ± SEM (see Table S5 for the values of the measurements). (c) Efficiency of Tyr, Phe, Trp, and Kyn quantification. Efficiency (EF) was calculated as follows: EF = ME × RE/100, where ME stands for the matrix effect (in %) and RE stands for recovery (in %). ME and RE were measured with the spiked 4 μM concentration of analytes. Data presented as mean ± SD, *n* = 3 (for details, see Table S4 and Figure S2).

In order to evaluate the Trp/Kyn ratio as a potential skin cancer biomarker for non-invasive diagnostics, robust biomarker sampling from the skin surface is needed. An optimal sampling technique for this particular biomarker should have capability to collect hydrophilic molecules, be nonirritant, and biocompatible. Targeting these general criteria, several different skin sampling techniques were selected for evaluation. Two of the chosen sampling techniques, tape stripping (TPS) and sampling with a hydrated cotton swab (CTN), directly collect biomarkers present on the skin surface. The other sampling techniques are based on 2 h, patch-like sampling with different hydrogels (agarose (AGR), chitosan (CHI), and agarose:chitosan mixture (AGC)) and hydrated starch films (STR). The 2 h sampling time was chosen based on our previous studies, which indicated that within 2 h of sampling, satisfactory amounts of analytes, for example, Trp and Kyn, can be collected for analytical measurements *in vitro*,^23^ and sufficient skin barrier hydration can be achieved within 1 h, facilitating skin permeability *in vivo*.^25^ In addition, sampling with chitosan hydrogel performed up to 8 h *in vivo* (Figure S3) indicated that the quantities of analytes collected positively correlated with the sampling time. Therefore, to ensure that adequate amounts of analytes were collected, and still keeping relatively short sampling times for practical reasons, 2 h was chosen as an optimal sampling time. The assessment of different sampling techniques was carried out by determining the quantities of Tyr, Phe, Trp, and Kyn and their ratios in the samples collected from the skin surface (Figure 2a,b). Additionally, the efficiency of LC–MS analysis (Figure 2c) was determined based on the matrix effect and recovery measurements for each sampling material (Table S4 and Figure S2).

As can be seen in Figure 2a, all sampling procedures collected biomarkers from the skin surface with the highest quantities being collected by STR. Sampling by CTN collected significantly lower amounts of Phe and Trp compared to all other sampling techniques (*p* < 0.001) and did not sample detectable amount of Kyn (see also Tables S5 and S6). However, CTN collected lower amounts of Tyr only compared to STR (*p* = 0.014) (Figure 2a). This, surprisingly, indicated that a 5 s sampling with CTN collects similar amounts of Tyr as sampling with the hydrogels for 2 h. Particularly, CTN samples contained 118 ± 127% (*n* = 48) Tyr, 28 ± 19% (*n* = 51) Phe, and 27 ± 19% (*n* = 51) Trp if compared to the total amount of these amino acids collected by 2 h sampling with hydrogels. The relatively higher amounts of CTN-collected Tyr might indicate that the abundance of this amino acid is higher on the surface of the skin. Regarding the effect of quantification efficiency on the differences observed between the sampling techniques, few points should be noted. First, in the case of STR, higher quantification efficiency was observed for Tyr. This might have slightly affected the fact that in the samples collected by STR, quantities of Tyr were higher. In addition, in the case of TPS, the quantification efficiency for this sampling material was highest for all analytes. Interestingly, quantification efficiency was lower for Tyr, in the case of CHI and AGC, and lower for STR and CTN in the case of Trp. Nevertheless, the differences observed between sampling techniques in terms of quantification efficiency were statistically significant only for Trp. Trp quantification efficiency was significantly higher for samples collected with TPS compared to CTN (*p* = 0.017) and STR (*p* = 0.042). This indicates that observed differences between the sampling quantities are mostly due to the ability of the material to sample from the skin surface.

There was no statistically significant difference between different hydrogels (AGR, AGC, and CHI) regarding their ability to collect the amino acids from the skin surface (Figure 2a, Tables S5 and S6). Positively charged CHI and non-charged AGR did not show difference, and hence, the charge on the polymer did not affect the sampling of these particular analytes. The quantities of analytes collected using hydrogels were 0.9 ± 0.9 nmol/cm^2^ (*n* = 48) of Tyr, 0.6 ± 0.4 nmol/cm^2^ (*n* = 51) of Phe, 0.4 ± 0.3 nmol/cm^2^ (*n* = 51) of Trp, and 0.02 ± 0.01 nmol/cm^2^ (*n* = 9) of Kyn (Table S5). In general, the amount of analytes collected using hydrogels or STR did not differ from the amount of analytes collected by TPS (Figure 2a). This suggests that sampling for 2 h pulls out the analytes form the skin depth of 1–3 μm (the thickness of the skin removed with three tape strips).^26^

Kyn was detected only in 21 out of total 102 samples collected from the skin surface. Specifically, Kyn was found if sampled with AGR (*n* = 8), AGC (*n* = 1), STR (*n* = 8), and TPS (*n* = 4). There was no statistically significant difference in the amount of Kyn collected by any of the techniques (see Figure 2, Table S5 and S6). It is important to note that the levels of detected Kyn were low (below LOQ), which means that the quantities of estimated Kyn might be inaccurate, and should be interpreted with the caution.

Furthermore, analysis of the data showed that the difference between the sampling techniques, in terms of amounts of analytes collected, was strongly attenuated if analyte ratios were considered. This can be easily discerned from Phe to Trp (Phe/Trp) and Phe/Tyr ratios in Figure 2b. For the Tyr/Trp and Phe/Tyr ratios, only CTN sampling technique showed a statistically significant difference, while for the Phe/Trp ratio there was no statistically significant difference between the investigated sampling techniques. Additionally, measurements of the skin resistance before and after the sampling have showed that all hydrogels and hydrated starch films applied on skin for 2 h equalized the physical skin barrier property among sampling sites and between individuals (Section S7). This means that most of the used sampling approaches reduced skin resistance variability, which might be beneficial in reducing variability of collected analytes due to more equalized fluxes across the skin.^27^

An important conclusion from this part of the study is that Phe/Trp and Phe/Tyr ratios show low variability, irrespective of the sampling approach. The Trp/Kyn ratio is an obvious biomarker, but skin surface accumulation of Kyn is very limited, and it might be difficult to quantify as shown in this study with healthy volunteers. In cancer cases when Trp is metabolized to Kyn, the concentration of Trp might be reduced and, thus, the Trp consumption can possibly be captured from the Phe/Trp ratio. Therefore, in addition to the Trp/Kyn ratio, the Phe/Trp ratio is of particular interest as a possible non-invasive skin cancer biomarker.

### Operating with Analyte Ratios Instead of Their Absolute Amounts Leads to Reduced Biomarker Variability

The absolute amounts of Tyr, Phe, Trp, and Kyn collected from the skin surface of healthy volunteers varied considerably (Figure S5 and Table S10). For example, on sampling by AGR, the coefficient of variation (% CV) between six individuals (*n* = 18 for AGR) was estimated to be 95, 68, and 61%, for Tyr, Phe, and Trp, respectively (Figure S5a and Table S10a). Usually, more than 50% CVs were found for absolute quantities sampled by other approaches, as summarized in Figure S5a. The variability of the amounts if compared for the same individual is lower; the CVs basically are below 50%, Figure S5b and Table S10b. This indicates that the sampling approaches are able to capture individual differences.

Assessment of the ratios between the analytes (Tyr/Trp, Phe/Trp, etc.), instead of the absolute quantities, noticeably attenuated the variation between (Figure S5c and Table S10a) and within (Figure S5d and Table S10b) individuals. Considering all hydrogel measurements, on average, the CV value between individuals in terms of absolute quantities was 79%; meanwhile, for their ratios, it decreased to 45%. Similarly, variation within individuals was 42% for absolute quantities and 21% for the ratios. These results imply that using ratios instead of absolute quantities is beneficial to reduce both intra- and inter-individual variations. In general, assessment of the ratios instead of absolute quantities decreases variability due to improved technical reproducibility, and reduced biological variation. It could be noted that metabolic homeostasis might be disturbed due to diet^28^ and disorders.^29^ However, under healthy conditions, we should expect a strong correlation between amino acids and their metabolites, that is, between Phe and Tyr and between Trp and Kyn.

**Figure 3:**
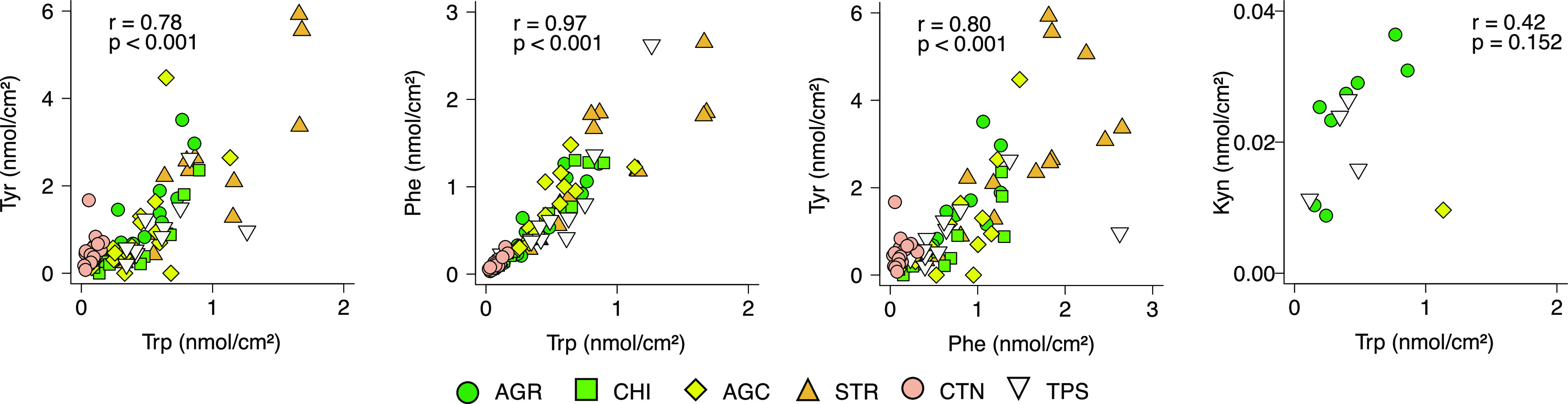
Spearman’s rank correlation between Tyr and Trp, Phe and Trp, Tyr and Phe, and Kyn and Trp. The correlation analysis was carried out for pooled analyte quantities estimated in samples collected by different sampling techniques, AGR, CHI, AGC, STR, CTN, and TPS.

Spearman’s correlation between the amino acids presented in Figure 3 showed that the strongest positive correlation was between Phe and Trp (*r* = 0.97 and *p* < 0.001), with a weaker correlation between Tyr and Phe (*r* = 0.80 and *p* < 0.001) and Tyr and Trp (*r* = 0.78 and *p* < 0.001) and no correlation between Kyn and Trp (*r* = 0.42 and *p* = 0.152). The lower correlation between Tyr and Phe and between Tyr and Trp compared to Phe and Trp could partly be due to the error in Tyr quantification (Tyr is the least stable compound). The absence of correlation between Kyn and other amino acids, especially Trp, could be due to several reasons. The composition of free amino acids in the SC has been shown to be similar to their composition in filaggrin.^30^ It is likely that Phe, Trp, and Tyr collected from the skin originate from degraded filaggrin and act as NMF. However, Kyn is not a part of NMF. The main sources of Kyn on/in the skin are Trp metabolism in skin^31^ and its diffusion from deeper skin layers, that is, from the blood/interstitial fluid and/or metabolic activity of skin microbiota.^11^ The absence of correlation between Kyn and the other amino acids should, however, be considered as a very preliminary observation due too low numbers of Kyn detection.

In conclusion, the lowest intra- and inter-personal CVs (Figure S5) and the highest Spearman’s correlation were found for the Phe/Trp ratio (Figure 3), suggesting that Phe/Trp might be a robust biomarker. The ratio might be a particularly important skin disorder biomarker because it accounts for Trp, a well-known source of Kyns, that is, metabolites involved in a plethora of anti-/pro-inflammatory and immune tolerance reactions.^32^

### Effect of Sweating on Sampling of Tyr, Phe, Trp, and Kyn on the Surface of Skin

**Figure 4:**
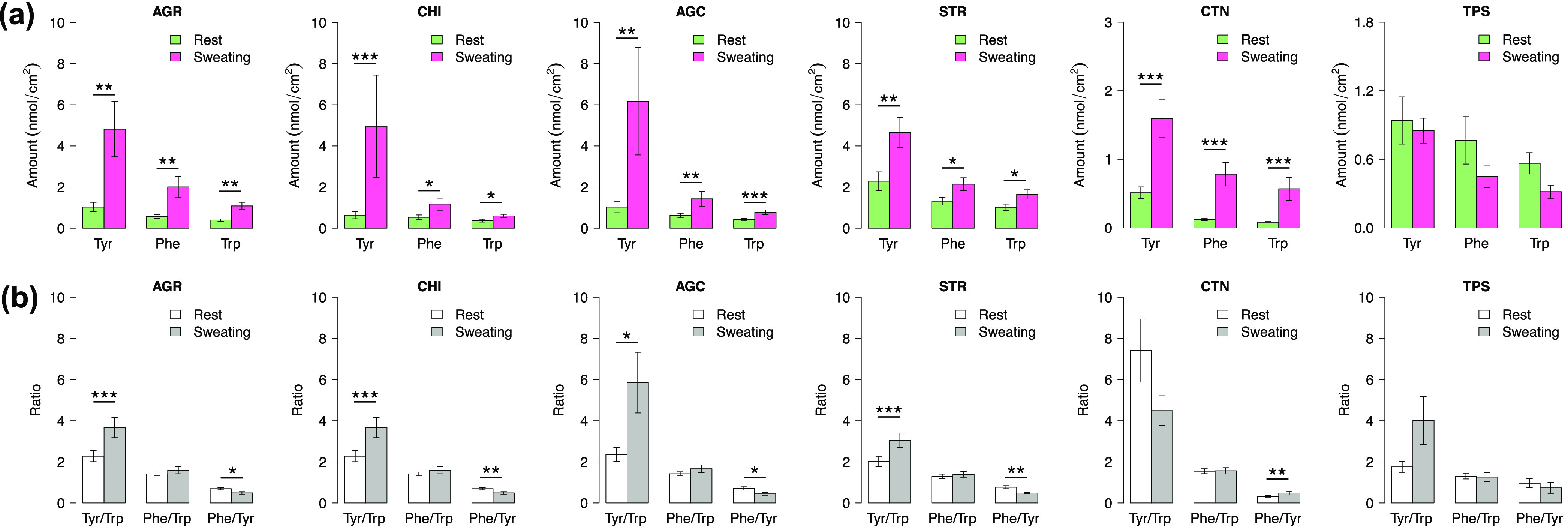
Influence of sweating on non-invasive sampling of Tyr, Phe, and Trp on the skin surface. Effect of sweating on the amino acid quantities (a) and their ratios (b) evaluated for each sampling technique. Data represent mean ± SEM (see Table S5 for the number of observations).

In order to exploit Tyr/Trp, Phe/Trp, Phe/Tyr, and Trp/Kyn ratios as robust biomarkers on skin, it is very important to understand factors that affect these quantities. As already mentioned, one significant source of the investigated amino acids is the NMF reservoir in the SC. Another source of amino acids is sweat. The precursor of sweat is the extracellular fluid, which means that many components found in sweat, including amino acids, originate from blood.^33^ Some compounds, however, enter the sweat as a result of production by eccrine glands. Keeping in mind the compositional complexity of sweat, we attempted to investigate if sweating has a tangible effect on sampling of the Tyr, Phe, Trp, and Kyn from the skin surface. The sampling was performed during extensive sweating using the same techniques and the same sites summarized in Table 1 and Figure 1, respectively. The comparison between the amino acids collected from the skin surface at rest and while sweating is shown in Figure 4.

As can be seen in Figure 4, the comparison of the biomarkers at rest and while sweating concerns only Tyr, Phe, and Trp because no Kyn was detected in any of the samples collected from the skin while sweating. The absence of Kyn could be due to low concentration; sweat samples were not pre-concentrated, owing to high concentrations of the other analytes. Figure 4a clearly shows that all sampling techniques, except TPS, collected significantly higher quantities of all three analytes, that is, Tyr, Phe, and Trp, while sweating compared to collection at rest. It is likely that sweat, consisting mostly of water, has a capacity to replenish skin NMF to some extent, and this is the reason for lower analyte collection by TPS after sweating (Figure 4a). This agrees with the results reported by Dunstan et al., 2016,^34^ which demonstrated that the concentration of amino acids decreases with sweating time and reaches blood levels after 30 min of sweating. Although, it cannot be excluded that the lower quantities of Tyr, Phe, and Trp collected by TPS after sweating are related to low amount of SC removed by tape striping of well-hydrated skin.

The quantities of collected Tyr were affected by sweating to the highest extent for all sampling techniques, except TPS. It could be due the highest difference of Tyr abundance in NMF versus in blood. Compared to Trp and Phe, Tyr is a more abundant NMF component in SC^35,36^ and has a higher presence in filaggrin.^24^ Specifically, the amount of Tyr in the SC exceeds Phe and Trp with factors of 2.5 and 2.4, respectively.^36^ In blood, the quantity of Tyr is just slightly higher; 1.3 times higher than Trp and 1.4 times higher than Phe.^37^ Therefore, it is likely that sweat “washes” out the SC reservoir of NMF and imposes the concentrations found in blood plasma.^33^

The ratios involving Tyr (e.g., Tyr/Trp and Phe/Tyr) determined in the samples collected from the skin surface during sweating were significantly different from the corresponding ratios sampled at rest (Figure 4b). However, there was no statistically significant difference between the Phe/Trp ratio estimated in the samples collected from the skin surface at rest or while sweating for all sampling techniques (Figure 4b). This result leads us to a very important conclusion. The comparison of topical collection of the amino acids during sweating versus at rest indicates that the Phe/Trp ratio is not significantly affected by sweating. This, once again, suggests that the Phe/Trp ratio deserves to be assessed as a possible and robust skin cancer biomarker.

### Comparison of the Amino Acid Ratios Collected from the Skin Surface at Rest, when Sweating, and in Blood Plasma

To relate topically sampled amino acid ratios to their systemic ratios, blood samples were analyzed. The concentrations of the analytes estimated in blood were as follows: 66.2 ± 16.5 μM Tyr, 46.9 ± 9.8 μM Phe, 58.5 ± 11.6 μM Trp, and 1.5 ± 0.2 μM Kyn (*n* = 6, Table S12). The concentrations of the analytes in blood plasma were in good agreement with the values reported for 100 healthy volunteers [90.6 ± 22.9 μM Tyr, 65.2 ± 11.1 μM Phe, 67.4 ± 10.2 μM Trp, and 1.8 ± 0.4 μM Kyn (*n* = 100)]^37^ (for further discussion on the analysis of the quantities of amino acids in blood, see Section S8). In general, the levels of amino acids were within the normal (healthy) plasma ranges as reported in the literature^36−38^ (Table S13) and in the Human Metabolome Database. Owing to incomparable volumes, a comparison between the absolute quantities of amino acids collected from the skin surface versus corresponding blood concentrations is irrelevant; instead, a comparison is carried out for the amount’s ratios. The ratios Tyr/Trp, Phe/Trp, and Phe/Tyr determined in samples collected from the skin surface at rest, skin surface while extensively sweating, and blood plasma are summarized in Table 2. The similarity between Tyr/Trp, Phe/Trp, and Phe/Tyr ratios estimated in the samples collected from the skin surface at rest and skin surface while sweating versus blood plasma probably can be referred to homeostasis in the overall, healthy human body, including the extensive skin organ (for further discussion, see Section S9). Since only healthy volunteers participated in the study, it is hard to anticipate how abundance and ratios of the amino acids in blood versus on the skin would be changed under disease conditions. For example, a study performed on psoriatic patients has found a correlation between the severity of the disease and the abundance of choline and citrulline in skin and in blood.^14^ However, the correlation between choline versus psoriasis severity was much stronger for samples collected from the skin surface (Spearman’s *r* = 0.785 and *p* < 0.0001) compared to those from blood (Spearman’s *r* = 0.347 and *p* = 0.0018).^14^ Similarly, a significant effect of treatment was observed for choline abundance measured in the samples collected from the skin surface but not in the blood. Projecting this into a situation of skin cancer, one would expect that at early skin cancer stages, metabolic changes are expected to occur within the tumor microenvironment (TME). Thus, locally altered skin chemistry should be easier to capture on skin than in the blood.

**Table 2:** Ratios Tyr/Trp, Phe/Trp, Phe/Tyr, and Trp/Kyn Estimated on the Skin Surface at Rest, Skin Surface While Excessively Sweating, and Blood Plasma Samples^a^

		non-invasive skin surface sampling						systemic
ratio	state	agarose (AGR)	chitosan (CHI)	AGR/CHI (AGC)	starch (STR)	cotton swab (CTN)	TPS	plasma (PLS)
Tyr/Trp	rest	2.3 ± 1.1	1.7 ± 0.9	2.4 ± 1.4	2.0 ± 1.0	7.4 ± 6.5	1.8 ± 0.9	1.1 ± 0.2
		(*n* = 18)	(*n* = 14)	(*n* = 16)	(*n* = 17)	(*n* = 18)	(*n* = 11)	(*n* = 6)
	sweating	3.7 ± 2.0	5.7 ± 6.5	5.9 ± 6.3	3.0 ± 1.4	4.5 ± 3.0	4.0 ± 3.7	
		(*n* = 17)	(*n* = 18)	(*n* = 18)	(*n* = 17)	(*n* = 17)	(*n* = 10)	
	CI (95%)	[0.4–2.4]	[0.0–8.2]	[0.3–8.1]	[1.4–3.6]	[1.8–10.2]	[0.6–5.0]	[0.9–1.3]
Phe/Trp^b^	rest	1.4 ± 0.4	1.4 ± 0.3	1.4 ± 0.4	1.3 ± 0.4	1.5 ± 0.5	1.3 ± 0.4	0.8 ± 0.1
		(*n* = 18)	(*n* = 15)	(*n* = 18)	(*n* = 17)	(*n* = 18)	(*n* = 11)	(*n* = 6)
	Sweating	1.6 ± 0.7	1.7 ± 0.7	1.7 ± 0.8	1.4 ± 0.6	1.6 ± 0.6	1.3 ± 0.7	
		(*n* = 17)	(*n* = 18)	(*n* = 18)	(*n* = 17)	(*n* = 17)	(*n* = 11)	
	CI (95%)	[1.1–1.5]	[1.0–2.0]	[1.0–2.0]	[0.9–1.7]	[1.2–2.0]	[0.8–1.8]	[0.7–0.9]
Phe/Tyr	rest	0.7 ± 0.2	1.1 ± 0.7	0.7 ± 0.3	0.8 ± 0.3	0.3 ± 0.2	1.0 ± 0.7	0.7 ± 0.1
		(*n* = 18)	(*n* = 14)	(*n* = 16)	(*n* = 17)	(*n* = 18)	(*n* = 11)	(*n* = 6)
	sweating	0.5 ± 0.2	0.4 ± 0.1	0.4 ± 0.3	0.5 ± 0.1	0.5 ± 0.4	0.7 ± 0.8	
		(*n* = 17)	(*n* = 18)	(*n* = 18)	(*n* = 17)	(*n* = 16)	(*n* = 9)	
	CI (95%)	[0.4–0.8]	[0.3–1.1]	[0.3–0.9]	[0.4–0.8]	[0.2–0.6]	[0.3–1.5]	[0.6–0.8]
Trp/Kyn	rest	17.7 ± 7.2		177.8	41.9 ± 20.7		17.7 ± 9.0	38.8 ± 5.1
		(*n* = 8)		(*n* = 1)	(*n* = 8)		(*n* = 4)	(*n* = 6)
	sweating							
	CI (95%)							[34.7–42.9]

a Results obtained under both biological conditions, at rest and while sweating, are presented separately. The values shown in square brackets report a 95% confidence interval [CI (95%)] for ratios collected at rest and while sweating.

b CI reported in the abstract is calculated by taking into account all sampling techniques.

### Possible Clinical Relevance for Monitoring Trp/Kyn and Phe/Trp Ratios

To support the possible clinical relevance of the investigated, potential skin cancer biomarkers, preliminary experiments were performed with a cell-cultured 3D skin models. The aim was to assess Tyr/Trp, Phe/Trp, Phe/Tyr, and Trp/Kyn ratios in one of the models of cancerous skin. In our previous work, we have shown that reconstituted human epidermis treated with IFN-γ invokes Trp metabolism to Kyn. The Trp transformation is attributable to upregulated expression of the enzyme indoleamine 2,3-dioxygenase (IDO-1), leading to a decreased Trp/Kyn ratio.^31^ In the current study, a 3D model of skin dermis/epidermis was stimulated either with the pro-inflammatory cytokine IFN-γ or with UV-B radiation as the sunlight exposure model. Then, the quantities of analytes and the ratios of Tyr/Trp, Phe/Trp, Phe/Tyr, and Trp/Kyn were determined in the cell culture medium and compared to ratios present in the culture medium of unstimulated skin, that is, control (Table S15). Treatment of a 3D skin model with IFN-γ decreased the Trp/Kyn ratio 80–100 times versus control (i.e., without treatment with IFN-γ). Additionally, due to Trp depletion, Tyr/Trp and Phe/Trp ratios increased 20–30 times. These preliminary results provide evidence that skin cancer cases with upregulated IDO-1 may alter (i.e., considerably decrease) the Trp/Kyn ratio in the TME. In this case, elevated concentrations of Kyn may also decrease the Kyn quantification difficulties that were experienced in the samples collected on skin of healthy subjects. The *in vitro* data also suggest that as an alternative to the Trp/Kyn ratio, Tyr/Trp and Phe/Trp ratios are additional tentative skin cancer biomarkers. Importantly, the *in vitro* experiments with the 3D skin model showed no change in the ratios determined in samples collected after skin exposure to UV-B radiation, implying that factors such as UV-B radiation probably do not alter the Trp/Kyn ratio. However, it should be noted that 3D skin models lacked melanocytes.

### Study Limitations

One of the obvious limitations of this study is the small number of participants. By virtue of the low concentrations of Kyn (<LOQ), we have observed some false/interfering signals coming from some sampling materials. *In vitro* experiments with 3D skin models must be considered as preliminary but nonetheless important for future studies. Specifically, the 3D skin model experiments should include sampling of biomarkers from the SC side of the skin; in this work, we have sampled the relevant amino acids in cell culture medium, which models the TME but excludes biomarkers permeating through the SC. The analytical method used in this study suffers from poor precision, that is, on average, CV (%) = 20 ± 9% and mean ± SD, *n* = 20. According to the FDA and EMA guidelines written for bioanalytical method validation, it is recommended to have CV < 15%.^39^ Therefore, LC–MS/MS analysis used in this study should be improved for future applications by, for example, using isotopically labelled analogues of the analytes as internal standards.

## Conclusions

In this work, we studied the feasibility of non-invasive *in vivo* monitoring of possible skin cancer biomarkers. The sampling was performed using three hydrogels (made of agarose and/or chitosan), hydrated starch films, cotton swabs, and tape stripping. The chosen LMW biomarkers were the three amino acids Tyr, Phe, and Trp and the Trp metabolite Kyn.

All sampling techniques successfully collected LMW biomarkers from the skin surface; however, the quantities collected differed significantly. The hydrated starch film collected the highest quantities of analytes whereas sampling with a cotton swab resulted in the lowest amounts. There was no significant difference between the hydrogels in terms of the collected amounts of biomarkers. Averaging the quantities of analytes collected using the three hydrogels provided sampled amounts of 0.9 ± 0.9 nmol/cm^2^ (*n* = 48) for Tyr, 0.6 ± 0.4 nmol/cm^2^ (*n* = 51) for Phe, 0.4 ± 0.3 nmol/cm^2^ (*n* = 51) for Trp, and 0.02 ± 0.01 nmol/cm^2^ (*n* = 9) for Kyn (Kyn < LOQ). The low quantities of Kyn were expected because the study was performed on healthy volunteers.

High intra- and inter-personal variability observed in absolute quantities of collected analytes was considerably attenuated by determining ratios of analytes. Due to low abundance of Kyn, Kyn was detected only in a few samples collected at rest and not detected in samples taken while sweating. The Phe/Trp ratio appeared to be very stable and not affected significantly by sampling technique or sweating. The Phe/Trp ratio was 1.3 ± 0.4 and 1.3 ± 0.7 in samples at rest and under sweating conditions, respectively.

The possible clinical relevance of monitoring Tyr, Phe, Trp, and Kyn was modeled by simulation of skin cancer development in 3D cell-cultured epidermis/dermis. Treatment of the skin equivalents with IFN-γ was used to induce the KP. Monitoring of biomarkers in the skin model showed that the Trp/Kyn ratio decreased 80–100 times and that the Phe/Trp and Tyr/Trp ratios increased 20–30 times. These results, together with the high reproducibility in the estimation of the Phe/Trp ratio on healthy human skin, suggest that not only the Trp/Kyn but also the Phe/Trp ratio could be evaluated in clinics as a possible biomarker for non-invasive detection of skin cancers, which employs an immune escape mechanism based on consumption of Trp and production of immune cell-suppressing Kyn.

## Supporting Information Available

Detailed overview of the experimental section outlining sampling material preparation, blood sampling procedure, skin barrier assessment by EIS, details regarding LC–MS/MS analysis and 3D cell-cultured skin models, matrix effect, recovery and analyte’s quantification efficiency determined for investigated sampling materials, effect of the sampling techniques on skin resistance, quantities of analytes and their ratios in blood versus skin surface at rest and while sweating, and brief literature overview on quantities of analytes (and their ratios) reported in the literature (PDF)
