# Supplementary material for: Non-Invasive, Topical Sampling of Potential, Low-Molecular Weight, Skin Cancer Biomarkers: A Study on Healthy Volunteers
Source: Anal Chem. 2022 Apr 8;94(15):5856–65. doi: 10.1021/acs.analchem.1c05470 (PMC9022073; doi:10.1021/acs.analchem.1c05470)
Supplement: Supplementary file 1 — ac1c05470_si_002.pdf [file ac1c05470_si_002.pdf]

SUPPORTING INFORMATION

Non-invasive, topical sampling of potential, low-molecular weight, skin cancer biomarkers: study on healthy volunteers

Skaidre Jankovskaja[1,2,‡,*], Maxim Morin[1,2, ‡], Anna Gustafsson[1,2], Chris D. Anderson[3,4], Boglarka Lehoczki[1,2], Johan Engblom[1,2], Sebastian Björklund[1,2], Melinda Rezeli[5], György Marko-Varga[5], and Tautgirdas Ruzgas[1,2,*]

[‡]These authors contributed equally
[1]Department of Biomedical Science, Malmö University, Sweden
[2]Biofilms - Research Center for Biointerfaces, Malmö University, Sweden
[3]Department of Biomedical and Clinical Sciences, Linköping University, Sweden
[4]Department of Dermatology and Venereology, Linköping, Sweden
[5]Clinical Protein Science and Imaging, Department of Biomedical Engineering, Lund University, Sweden

Contents

Experimental section 3

Fig. S1. A custom-made frame from Chemotechnique skin patch 3

Section S1. Chemicals 3

Section S2. Sampling materials preparation 3

Table S1. Weight of starch films measured before and after hydration 4

Section S3. Blood sampling 5

Section S4. Skin barrier assessment by EIS and TEWL measurements 5

Table S2. MRM parameters for LC-MS/MS analysis 5

Table S3. Analytical performance of LC-MS/MS method for analysis of Tyr, Phe, Trp and Kyn 6

Section S5. Preparation of the standard solutions of Tyr, Phe, Trp and Kyn 6

Table S4. Matrix effect, recovery and efficiency determined for investigated sampling techniques. 6

Fig. S2. Comparison of the influence of matrix effect, revovery and efficiency of different sampling materials on analytes quantification 9

Section S6. 3D cell culture model of human dermis/epidermis 9

Results and discussion 9

Fig. S3. Sampling of analytes from the skin surface as a function of time 9

Table S5. Quantity of the analytes collected by different sampling techniques 10

Table S6. P-values from sampling approach comparison. 11

Table S7. TEWL measured before and after tape strips 12

Section S7. Effect of different sampling techniques on skin resistance 12

Fig. S4. Skin resistance measured before and after sampling 13

Table S8. The effect of the sampling techniques on the skin resistance 13

Table S9. Weight change of sampling material after 2 h of sampling 13

Table S10. CV's (%) for the quantity of analytes and their ratios estimated in the samples collected from the skin surface by different sampling techniques 14

Fig. S5. Comparison of the CV's (%) determined for the absolute quantity of analytes and their corresponding ratios in the samples collected from the skin surface by different sampling techniques. 14

Fig. S6. Comparison between the quantity of analytes and the corresponding ratios in samples collected close to wrist and elbow 15

Table S11. Physicochemical characteristics of Tyr, Phe, Trp and Kyn 15

Table S12. Blood plasma concentration of Tyr, Phe, Trp, and Kyn 16

Section S8. Discussion about Tyr, Phe, Trp and Kyn concentration in blood, sweat and skin surface 16

Table S13. Literature values of Tyr, Phe, Trp, and Kyn measured in blood, skin surface, and sweat 17

Section 9. Comparison of the ratios of analytes collected from skin surface at rest, when sweating, and in blood plasma 18

Fig. S7. The ratios of Tyr/Trp, Phe/Trp, and Phe/Tyr in samples collected from the skin surface at rest, when sweating, and in blood plasma 18

Table S14. Comparison between the ratios in blood vs skin surface (P-values) 19

Table S15. The effect of IFN-γ and UV-B radiation treatment of skin model on Tyr/Trp, Phe/Trp, Tyr/Phe, and Trp/Kyn ratios 19

References 20

Experimental section

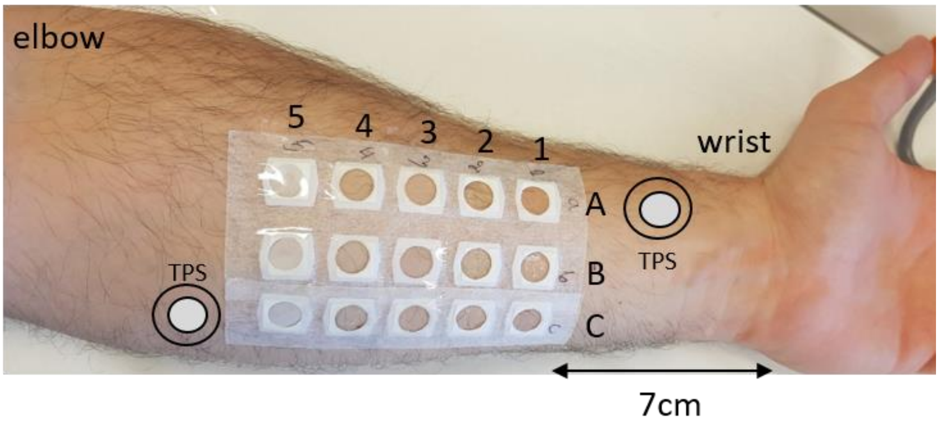

Fig. S1. A custom-made frame from Chemotechnique skin patch (Chemotechnique MB Diagnostics AB, Vellinge, Sweden) used for defining sampling sites. A, B, C indicates placement of the same material or same procedure of sampling. 1, 2, 3, 4 and 5 indicates random placement of sampling material. TPS stands for tape sampling technique which was performed on two locations: close to wrist or elbow.

Section S1. Chemicals

Trp (L-tryptophan) and Kyn (L-kynurenine), reagent grade > 98% (HPLC) were purchased from Sigma-Aldrich (St. Louis, MO, USA). Tyr (L-Tyrosine) with 99% purity was purchased from Alfa Aesar (ThermoFisher GmbH, Kandel, Germany) and Phe (L-phenylalanine) with 99% purity was purchased from Lancaster (White Lund, England). Methanol (MeOH), HPLC gradient grade, was obtained from VWR International (Fontenay-sous-Bois, France). Formic acid (> 99%) was purchased from Merck (Darmstadt, Germany). Acetic acid (>99%), sodium azide (NaN_3), high molecular weight chitosan and BioReagent, low electroendosmosis (EEO) agarose were purchased from Sigma-Aldrich (St. Louis, MO, USA). Potato starch films were kindly provided by Magle ChemoSwed (Malmö, Sweden). Tape strips (D-squame® D100, round tape, d = 22 mm) and standardized pressurizer (D500-D-squame® pressure instrument) were obtained from CuDerm (CuDerm, Dallas TX, USA). Cotton swabs (Såklart, Axfood, Sweden) were purchased at a local store.

Section S2. Sampling material preparation

Chitosan gel (CHI) 2 wt % was prepared by dissolving chitosan in 1 % (v/v) acetic acid aqueous solution with pH adjusted to 5.0 and left under vigorous stirring overnight. Agarose hydrogel (AGR) 2 wt % was prepared by mixing 200 mg of agarose with 9.8 mL of Milli-Q water and heating it for a total of 2 min in the microwave, while stirring periodically, until agarose was completely dissolved. Then, 10 mL of hot agarose solution was poured into a (Ø 90 mm) Petri dish and left to cool down at room temperature for 10-15 min. Once the AGR solidified, the Petri dish was sealed with tape and stored at +4 °C until use (not longer than 16 h). At the day of experiment, circular pieces (ca. 10 mm diameter; 90 ± 11 mg (mean ± SD; n = 36)) were punched out from the AGR gel. The combination of CHI and AGR (AGC) hydrogel was prepared by mixing 200 mg of agarose and 200 mg of chitosan in 9.6 mL of 1 % v/v acetic acid solution (pH 5.0) overnight. After that AGC mixture was heated up in the microwave for 2 min, while mixing it with a spatula every 10-20 s. Once the AGC mixture dissolved, the same procedure as for AGR was followed to prepare circular pieces (ca. 10 mm diameter; 103 mg ± 18 mg (mean ± SD; n = 36)). Potato starch films were hydrated for 1 hour in Milli-Q water containing 10 % v/v of glycerol resulting in a final water content of 55 ± 5 wt % (mean ± SD, n = 36) (**Table S1**).

Table S1. Weight of starch films measured before and after hydration. Starch film (area 0.785 cm^2) hydration was done by immersing it into 1 mL solution containing 90% water and 10% glycerol and shaking for 1 h.

No.	Weight (mg)			
	Initial	After hydration	Difference	Weight change (%)
1	16.7	39.7	23.0	57.9
2	17.9	44.9	27.0	60.1
3	18.6	42.6	24.0	56.3
4	15.8	37.6	21.8	57.9
5	18.3	44.9	26.6	59.2
6	15.5	36.2	20.7	57.1
7	16.5	38.5	22.0	57.1
8	14.6	31.8	17.2	54.2
9	15.3	34.6	19.3	55.7
10	21.8	47.8	26.0	54.4
11	19.9	45.9	26.0	56.7
12	20.6	48.4	27.8	57.4
13	18.7	43.4	24.7	56.9
14	19.3	43.4	24.1	55.5
15	20.2	37.2	17.1	45.8
16	21.8	51.5	29.7	57.7
17	19.2	44.6	25.4	57.0
18	18.3	42.0	23.7	56.4
19	14.1	33.5	19.4	57.9
20	14.7	22.5	7.8	34.6
21	17.2	41.3	24.1	58.3
22	16.1	37.2	21.1	56.7
23	14.7	31.2	16.5	52.9
24	14.8	33.4	18.6	55.8
25	14.1	22.3	8.3	37.0
26	18.6	43.6	25.0	57.3
27	17.8	42.3	24.5	58.0
28	21.0	45.8	24.9	54.3
29	16.0	36.3	20.3	56.0
30	20.9	47.0	26.1	55.5
31	20.8	47.2	26.4	55.8
32	20.5	45.3	24.8	54.7
33	20.9	46.8	25.9	55.3
34	20.9	46.4	25.5	54.9
35	16.2	40.0	23.8	59.6
36	18.2	42.2	24.0	56.8
**Mean**	**18.0**	**40.5**	**22.6**	**55.1**
**SD**	**2.4**	**6.8**	**4.8**	**5.3**
**CV (%)**	**13.5**	**16.7**	**21.1**	**9.7**

Section S3. Blood sampling

Prior to blood collection, the participants had refrained from eating for 12 hours. Blood samples were collected in the morning after 15 min of rest. Approximately 4 mL blood was collected into lithium heparin-coated tubes (Becton Dickinson and Company, BD). All tubes were kept in an upright position at room temperature for 30 min where upon plasma was separated by 10 min centrifugation at 2000 × g. After centrifugation, 0.5 mL plasma aliquots were immediately frozen and stored at -80 °C. Prior to analysis, plasma proteins were precipitated by adding 0.4 mL of plasma to 1.2 mL of ice-cold methanol. The sample was vortexed and left on ice for 1 h. After that, plasma samples were centrifuged at 14,000 × g for 10 min at 5 °C. Following centrifugation, the supernatant was collected and solvents were removed using a centrifugal evaporator, and the residue was re-suspended in 1.6 mL of 20% (v/v) MeOH in Milli-Q water.

Section S4. Skin barrier assessment by EIS and TEWL measurements

The physical barrier properties of the skin were assessed by means of transepidermal water loss (TEWL) and electrical impedance spectroscopy (EIS). TEWL measurements were performed using Delfin VapoMeter SWL 5658 (Vapometer®, Delfin Technologies, Finland) before and after tape sampling (3 tape strips in total) (Table S7). The barrier properties of SC were also investigated by means of EIS, which measures the opposition of material to the flow of alternating currents at various frequencies[1]. It is generally known that the EIS response of skin is dominated by the SC[2–4]. In the low frequency regime, (< 1 kHz), the EIS response reflects the resistive properties of the skin barrier governed by appendageal pathways (hair follicles and sweat ducts)[5], which is in turn related to the permeability of the skin barrier[6]. Therefore, EIS at low frequencies can be used for the assessment of the skin barrier integrity. In this work, the EIS response of skin was measured with Nevisens® (SciBase, Sweden) before and after sampling of biomarkers in order to investigate the influence of sampling material on skin impedance. The Nevisense device, originally developed as a tool for skin cancer diagnosis[7], has a spring-loading mechanism for controlling the pressure by which a five-bar electrode probe is pressed against the skin. The electrodes contain micro-pins (150 µm long). The impedance of skin is measured at four depth settings utilizing 10 permutations (due to the five-bar electrode) by applying a weak electrical current at 35 different frequencies (1 kHz – 2.5 MHz). The applied voltage and the resulting current are limited to 150 mV and 75 µA, respectively. The absolute impedance $|Z|$ was determined from the real (Z_{Re}) and imaginary (Z_{Im}) impedance components according to $|Z| = \sqrt{(Z_{\mathrm{Re}})^2 + (Z_{\mathrm{Im}})^2}$. After that, $|Z|$ at 1 kHz (kOhm) was averaged over all depths and used as an electrical resistance characteristic of the SC barrier, which from now on will be referred to as skin resistance. The change in skin resistance was then calculated as $100\% * (|Z|_{\mathrm{init}} - |Z|_{\mathrm{after}}) / (|Z|_{\mathrm{init}})$, in order to assess the influence of the sampling materials on the skin barrier.

Table S2. MRM parameters for LC-MS/MS analysis. List of chromatographic retention times (RT), molecular weights (MW), selected precursor and product ions for multiple reaction monitoring (MRM) scans, dwell time, cone voltage and collision energies (CE) for each analyte. Dwell time in all cases was 0.08 s.

Analyte	RT (min)	MW (Da)	Precursor ion	Precursor (m/z)	Product (m/z)	Cone (V)	CE (eV)
Tyrosine (Tyr)	7.4	181.19	$[M+H]^+$	182.1	165.3	30	10
				182.1	136.5	30	10
Kynurenine (Kyn)	8.6	208.22	$[M+H]^+$	209.3	192.3	10	20
				209.3	94.0	10	20
Phenylalanine (Phe)	9.4	165.19	$[M+H]^+$	166.2	120.3	20	20
				166.2	103.2	20	30
Tryptophan (Trp)	10.5	204.23	$[M+H]^+$	205.3	118.3	20	20
				205.3	144.3	20	20

Table S3. Analytical performance of LC-MS/MS method for analysis of Tyr, Phe, Trp and Kyn.

a) Precision of the quantification of the analytes. Inter-day precision of quantification of the analytical method. Calibration solutions were prepared for Tyr, Phe, Trp, and Kyn, and measured during three consecutive days. The precision is defined as the CV (%) of these measurements, i.e., CV = SD / Mean x 100%.

	Amount (pmol)		Inter-day precision (% CV)			
	Trp, Phe, Kyn	Tyr	Trp	Phe	Tyr	Kyn
< LOQ	0.937	3.75	*20*	-	-	*30*
	1.875	7.5	*31*	-	-	*13*
	3.75	15	*24*	*39*	*21*	*20*
	7.5	30	*28*	*39*	*23*	*6*
> LOQ	15	60	4	15	27	18
	30	120	13	19	27	18
	60	240	9	15	8	15
	120	480	14	23	19	32
	240	960	25	19	34	38
Precision > LOQ (mean ± SD, *n*=5)			13±8	18±4	23±10	24±10
Precision at LOQ			4	15	27	18

b) Limit of detection (LOD) and quantification (LOQ) of the analytical method. LOD and LOQ were determined based on linear regression analysis of the mean values of the calibration standards (*n* = 3). The LOD and LOQ were determined as follows: LOD = 3.3σ/slope and LOQ as LOQ = 10σ/slope, where σ is the standard error of the y-intercept from the regression analysis.

Parameter	Amount (pmol)			
	Trp	Phe	Tyr	Kyn
LOD	5.4	4.0	17.4	7.4
LOQ	16.5	12.1	52.8	22.6

Section S5. Preparation of the standard solution of Tyr, Phe, Trp and Kyn

Stock solutions of Trp, 4.08 mg/mL (20 mM), Kyn, 4.16 mg/mL (20 mM), and Phe, 1 mg/mL (6.05 mM) were prepared in Milli-Q water. A stock solution of Tyr was prepared by dissolving in 99.9 % formic acid and diluting it further with Milli-Q water to reach 1 mg/mL (5.52 mM) (final concentration of formic acid did not exceed 1 % v/v). Fresh stock solutions of Trp, Kyn and Phe were kept at -20 °C for no longer than 2 weeks and used to prepare dilution series for or LC-MS/MS calibration. Dilution series were prepared using 20 % (v/v) of MeOH in Milli-Q water. Due to Tyr instability and poor solubility, a fresh stock solution was prepared each time LC-MS/MS analysis was performed.

Table S4. Matrix effect, recovery and efficiency determined for investigated sampling techniques.

a) Matrix effect on Tyr, Phe, Trp and Kyn quantification. Blank extracts of different sampling materials were used to simulate matrix effect. Matrix effect (ME) was calculated as follows: ME=A/B*100%, where A stands for concentration of analyte measured in the sample prepared in extracted blank, and B stands for concentration of analyte measured in the sample prepared in Milli-Q water. ME presented as mean ± SD, *n*=3.

Material	Spiked concentrations	Matrix effect (%)			
		Tyr	Phe	Trp	Kyn
AGR	2 µM	78.6±25.0	79.5±22.2	77.1±24.9	79.9±20.2
	4 µM	85.2±28.6	85.3±28.4	84.5±28.9	85.8±25.8
CHI	2 µM	83.6±12.9	87.4±12.5	88.2±14.3	85.0±9.0
	4 µM	102.0±10.1	111.7±11.4	108.1±11.3	108.1±12.6
AGC	2 µM	84.7±20.5	88.4±19.2	90.3±24.0	89.2±18.3
	4 µM	84.3±12.3	88.9±13.6	88.8±13.6	88.8±12.1

STR	2 µM	119.4±33.0	96.4±20.7	85.9±16.5	90.4±13.6
	4 µM	109.1±18.8	96.0±15.7	83.8±11.9	86.1±10.8
CTN	2 µM	105.9±21.2	96.1±18.7	87.2±18.2	89.8±16.5
	4 µM	107.9±18.4	106.6±21.4	94.9±16.5	101.3±17.4
TPS	2 µM	102.6±2.2	106.6±2.9	102.6±3.4	97.7±1.4
	4 µM	116.5±3.1	106.6±3.2	115.9±3.7	112.2±2.7

b) Recovery of Tyr, Phe, Trp and Kyn. Known concentrations of analytes were spiked into a different sampling materials, incubated for 2 h and then extraction procedure for each sampling material was followed as described in experimental section of the article (see section *Collection and extraction of the biomarkers*). Recovery (RE) was calculated as follows: RE=C/B*100%, where C stands for concentration determined in the sample prepared by spiking the analyte before the extraction procedure, and B stands for concentration of analyte measured in the sample prepared in Milli-Q water. RE presented as mean±SD, *n*=3.

Material	Spiked concentrations	Recovery (%)			
		Tyr	Phe	Trp	Kyn
AGR	2 µM	122.1±10.2	118.6±7.7	117.7±11.8	105.0±5.6
	4 µM	99.0±5.5	93.0±5.5	92.6±5.5	87.5±0.4
CHI	2 µM	74.7±22.0	99.5±17.3	88.1±19.9	79.4±12.1
	4 µM	66.5±13.1	69.6±11.8	73.0±16.4	74.8±12.5
AGC	2 µM	103.1±18.3	113.3±13.7	102.9±16.8	96.8±8.7
	4 µM	84.1±11.2	82.5±10.5	78.4±10.9	92.1±8.6
STR	2 µM	130.7±16.6	113.6±9.7	105.3±12.4	94.8±5.2
	4 µM	102.1±8.4	91.6±8.3	70.8±3.4	83.9±3.4
CTN	2 µM	98.8±8.8	102.6±6.3	72.7±5.0	87.0±5.2
	4 µM	84.2±10.4	86.4±12.5	53.0±8.4	78.4±10.3
TPS	2 µM	130.3±39.7	132.1±25.8	135.2±31.6	106.6±19.9
	4 µM	98.5±14.2	91.6±11.8	98.2±12.6	89.8±10.8

c) Efficiency of Tyr, Phe, Trp and Kyn quantification. Efficiency (EF) was calculated as follows: EF=ME*RE/100, where ME stands for matrix effect (Table 4a), and RE stands for recovery (Table 4b). EF presented as mean±SD, *n*=3.

Material	Spiked concentrations	Recovery (%)			
		Tyr	Phe	Trp	Kyn
AGR	2 µM	122.1±10.2	118.6±7.7	117.7±11.8	105.0±5.6
	4 µM	99.0±5.5	93.0±5.5	92.6±5.5	87.5±0.4
CHI	2 µM	74.7±22.0	99.5±17.3	88.1±19.9	79.4±12.1
	4 µM	66.5±13.1	69.6±11.8	73.0±16.4	74.8±12.5
AGC	2 µM	103.1±18.3	113.3±13.7	102.9±16.8	96.8±8.7
	4 µM	84.1±11.2	82.5±10.5	78.4±10.9	92.1±8.6
STR	2 µM	130.7±16.6	113.6±9.7	105.3±12.4	94.8±5.2
	4 µM	102.1±8.4	91.6±8.3	70.8±3.4	83.9±3.4
CTN	2 µM	98.8±8.8	102.6±6.3	72.7±5.0	87.0±5.2
	4 µM	84.2±10.4	86.4±12.5	53.0±8.4	78.4±10.3
TPS	2 µM	130.3±39.7	132.1±25.8	135.2±31.6	106.6±19.9
	4 µM	98.5±14.2	91.6±11.8	98.2±12.6	89.8±10.8

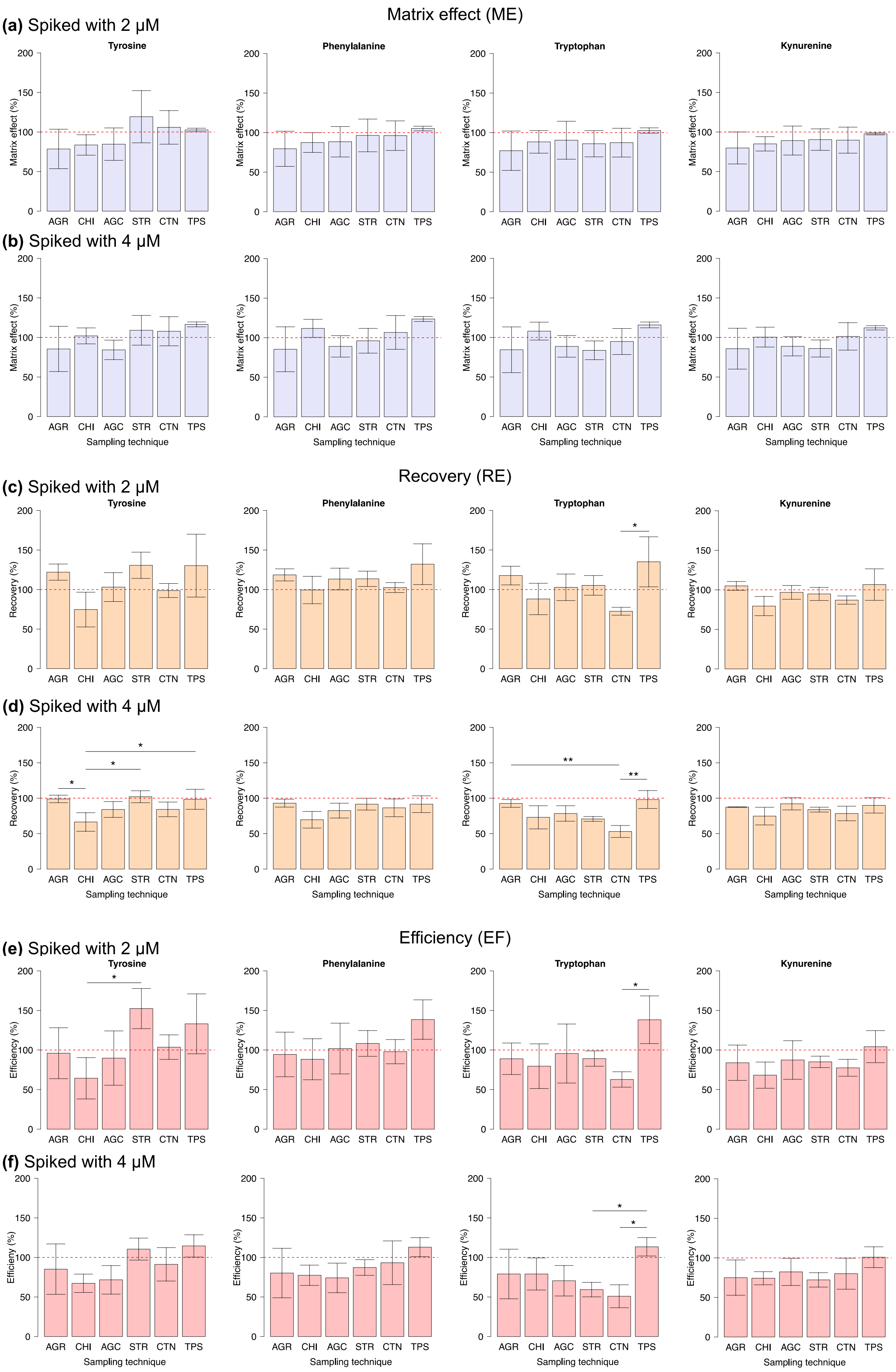
Matrix effect (ME)
(a) Spiked with 2 μM
Tyrosine
Phenylalanine
Tryptophan
Kynurenine
Matrix effect (%)
0
50
100
150
200
AGR CHI AGC STR CTN TPS
(b) Spiked with 4 μM
Sampling technique
Recovery (RE)
(c) Spiked with 2 μM
Recovery (%)
(d) Spiked with 4 μM
*
**
Efficiency (EF)
(e) Spiked with 2 μM
Efficiency (%)
(f) Spiked with 4 μM

Fig. S2. Comparison of the influence of matrix effect (ME) (a-b), recovery (RE) (c-d) and efficiency (EF) (e-f) on Tyr, Phe, Trp and Kyn quantification determined for each investigated sampling technique (see **Table S4** for ME, RE, and EF determination procedure description). Data is presented as mean±SD, n=3. Comparison between the sampling material's ME, RE, and EF was done by performing one-way Anova with Tukey test.

Section S6. 3D cell culture model of human dermis/epidermis

Cell culture

Primary human epidermal keratinocytes isolated from neonatal foreskin (HEKn, Thermo Fischer Scientific) were expanded in low-calcium serum-free EpiLife growth medium supplemented with 1 % of human keratinocyte growth supplement (HKGS) and 0.2 % gentamycin/amphotericin (Thermo Fischer Scientific). HKGS includes bovine pituitary extract, 0.2% v/v; bovine insulin, 5 µg/ml; hydrocortisone, 0.18 µg/ml; bovine transferrin, 5 µg/ml; human epidermal growth factor, 0.2 ng/ml. Cells were cultured at 37 °C in 5 % CO_2 in a humidified atmosphere. The growth medium was changed every other day until cells reached 50 % confluence, and every day thereafter. Primary human dermal fibroblasts isolated from neonatal foreskin (ATCC® CRL-2522™) were maintained in Eagle's Minimum Essential Medium (MEM) supplemented with 10 % fetal bovine serum (FBS) and 1 % penicillin/streptomycin (PEST, Thermo Fischer Scientific). Cells were cultured at 37 °C in 5 % CO_2 humidified atmosphere. The cells were passaged with trypsin-EDTA (0.05 %) at 75-80 % confluence.

In vitro 3D skin model

Dermal equivalents were prepared by mixing a solution of collagen type 1, extracted from rat tail tendons (Merck), with a buffer solution to achieve a final collagen concentration of 2.5 mg/ml. The solution was mixed thoroughly and 100 µl was added into Millicell® hanging cell culture insert (PET membrane diameter 12 mm, pore size 0.4 µm, Millipore) and polymerized in a humidified incubator at 37 ˚C 5 % CO_2 for 15 min. Then, 300 µl of a fibroblast suspension of 0.5 x 10^5 per ml collagen solution (2.5 mg/ml) was added to the inserts. After polymerization, MEM supplemented with 5 % FBS and 1 % PEST was added to each culture. The cultures were kept submerged for 7 days and the culture medium was changed every 2 days. HEKn at passage 2-3 were seeded on top of the contracted collagen gels. Culture medium was removed from the gels, keratinocytes (5 x 10^5/insert) were added and incubated at 37 ˚C, 45 min before addition of Epilife supplemented with HKGS (1%), ascorbic acid (50 µg/ml), and gentamycin/amphotericin (0.2 %). After 48 h, the cells were lifted to the air-liquid interface (ALI) by removing medium from the upper part of the membrane and the new culture medium was additionally supplemented with Ca^{2+} (1.5 mM). The inserts were then maintained at the ALI for 14 days, replacing the culture medium every other day.

Results and discussion

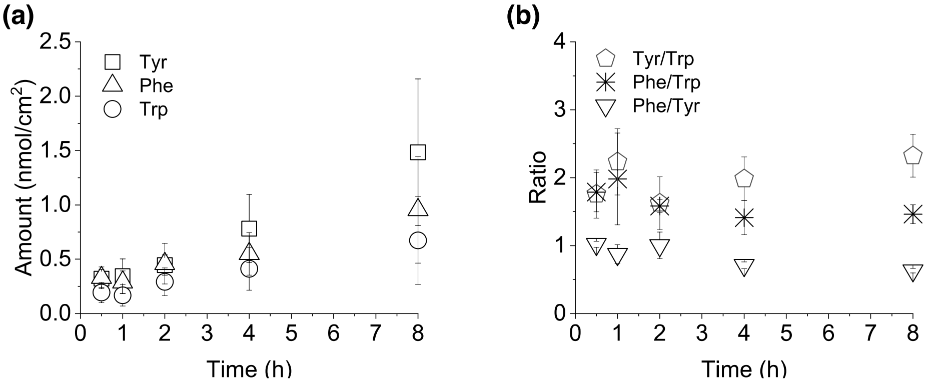

Fig. S3. Sampling of tyrosine (Tyr), phenylalanine (Phe), and tryptophan (Trp), using chitosan (CHI) hydrogel, from the skin surface over time. For each time point, the hydrogel was applied on the skin surface at 3 adjacent locations, *i.e.*, A, B, C as shown in **Fig. S1**, on the same individual. (a) Cumulative amount of Tyr, Phe, and Trp over 8 h sampling time. (B) Ratios between the analytes as a function of time. The Tyr/Trp and Phe/Trp ratios were not statistically different over the different sampling time. The Phe/Tyr ratio was significantly higher for samples collected over 0.5 h, as compared to 4 h (p=0.045) and 8 h (p=0.012); Phe/Tyr ratio determined in samples collected over 2 h was significantly higher compared to 8 h (p=0.016). Comparison between amounts and ratios was done by performing one-way Anova with multiple testing (Tukey test). The figure was adopted from our recently published work[8].

Table S5. Quantity of the analytes collected by different sampling techniques. Sampling was performed on skin surface of six healthy volunteers, 18 sampling sites, at rest (*i.e.*, Rest) and while sweating (*i.e.*, Sweating). ND – not detected.

Sampling technique	Analyte	Collected amount (nmol/cm^2)	
		Rest	Sweating
Agarose (AGR)	Tyr	1.0±1.0 (n=18)	4.8±5.5 (n=17)
	Phe	0.6±0.4 (n=18)	2.0±2.1 (n=17)
	Trp	0.4±0.2 (n=18)	1.1±0.7 (n=17)
	Kyn	0.02±0.01 (n=8)	ND
Chitosan (CHI)	Tyr	0.6±0.7 (n=14)	5.0±10.5 (n=18)
	Phe	0.5±0.4 (n=15)	1.2±1.2 (n=18)
	Trp	0.4±0.3 (n=15)	0.6±0.3 (n=18)
	Kyn	ND	ND
Chitosan+ Agarose (AGC)	Tyr	1.0±1.1 (n=16)	6.2±11.1 (n=18)
	Phe	0.6±0.4 (n=18)	1.4±1.5 (n=18)
	Trp	0.4±0.3 (n=18)	0.8±0.5 (n=18)
	Kyn	0.01 (n=1)	ND
Starch (STR)	Tyr	2.3±1.9 (n=17)	4.6±3.0 (n=17)
	Phe	1.3±0.8 (n=17)	2.1±1.3 (n=17)
	Trp	1.0±0.7 (n=17)	1.6±0.9 (n=17)
	Kyn	0.04±0.01 (n=8)	ND
Cotton swab (CTN)	Tyr	0.5±0.4 (n=18)	1.6±1.1 (n=17)
	Phe	0.1±0.1 (n=18)	0.8±0.7 (n=17)
	Trp	0.1±0.04 (n=18)	0.6±0.1 (n=18)
	Kyn	ND	ND
Tape sampling (TPS)	Tyr	0.9±0.7 (n=11)	0.9±0.3 (n=10)
	Phe	0.8±0.7 (n=11)	0.5±0.3 (n=11)
	Trp	0.6±0.3 (n=11)	0.3±0.2 (n=12)
	Kyn	0.02±0.01 (n=4)	ND

Table S6. *p*-values from sampling approach comparison. *p*-values derived from the statistical analysis performed for the comparison of sampling approaches using the mean analyte quantities and their ratios. Pairwise comparison between the quantity of the analytes, collected by different sampling techniques, was done by using Kruskal Wallis tests with Wilcoxon rank sum test and false discovery rate correction. Analyte ratios determined in the samples collected by different sampling techniques were compared by performing one-way Anova with Tukey test. The rounded numbers 0.000 refer to $p < 0.0001$.

a) Tryptophan (Trp)

	AGR	AGC	CHI	CTN	STR
AGC	0.864	-	-	-	-
CHI	0.595	0.595	-	-	-
CTN	0.000	0.000	0.000	-	-
STR	0.001	0.002	0.002	0.000	-
TPS	0.152	0.152	0.186	0.000	0.136

b) Phenylalanine (Phe)

	AGR	AGC	CHI	CTN	STR
AGC	0.767	-	-	-	-
CHI	0.702	0.501	-	-	-
CTN	0.000	0.000	0.000	-	-
STR	0.005	0.016	0.004	0.000	-
TPS	0.501	0.702	0.356	0.000	0.136

c) Tyrosine (Tyr)

	AGR	AGC	CHI	CTN	STR
AGC	1.000	-	-	-	-
CHI	0.213	0.213	-	-	-
CTN	0.213	0.213	0.928	-	-
STR	0.102	0.174	0.014	0.014	-
TPS	0.854	0.854	0.212	0.174	0.213

d) Kynurenine (Kyn)

	AGR	AGC	CHI	CTN	STR
AGC	0.562	-	-	-	-
CHI	-	-	-	-	-
CTN	-	-	-	-	-
STR	0.086	0.092	-	-	-
TPS	0.884	0.827	-	-	0.053

e) Tyr/Trp

	AGR	AGC	CHI	CTN	STR
AGC	1.000	-	-	-	-
CHI	0.992	0.988	-	-	-
CTN	0.000	0.000	0.000	-	-
STR	1.000	0.999	0.999	0.000	-
TPS	0.998	0.996	1.000	0.000	1.000

f) Phe/Trp

	AGR	AGC	CHI	CTN	STR
AGC	1.000	-	-	-	-
CHI	0.999	0.999	-	-	-
CTN	0.929	0.942	0.798	-	-
STR	0.970	0.961	0.999	0.516	-
TPS	0.982	0.976	0.999	0.643	1.000

g) Phe/Tyr

	AGR	AGC	CHI	CTN	STR
AGC	1.000	-	-	-	-
CHI	0.147	0.192	-	-	-
CTN	0.082	0.085	0.000	-	-
STR	0.996	0.998	0.378	0.024	-
TPS	0.572	0.635	0.999	0.002	0.845

j) Trp/Kyn

	AGR	AGC	CHI	CTN	STR
AGC	0.000	-	-	-	-
CHI	-	-	-	-	-
CTN	-	-	-	-	-
STR	0.017	0.000	-	-	-
TPS	1.000	0.000	-	-	0.060

Table S7. Trans epidermal water loss (TEWL) measured on skin at one location per individual before tape strips (baseline) and after three tape strips. The baseline TEWL values measured on the study participants were in the healthy skin range[9]. TEWL measurement after tape stripping did not indicate any skin barrier damage, *i.e.*, no significant changes in TEWL values were observed.

Individual	TEWL ($g/m^2 \cdot h$)		
	Baseline	After 3 tape strips	Change (%)
01F	11.8	11.4	-3
02F	11.1	11.3	2
03F	15	20.8	39
01M	9.8	11.4	16
02M	11.9	11.9	0
03M	10.8	13.4	24

Section S7. Effect of different sampling techniques on skin resistance

The electrical resistance of the SC is related to physical barrier properties of the skin membrane[6]. Therefore, EIS measurements are suitable to assess influence of the sampling technique on the skin barrier. This was investigated by measuring EIS responses of skin prior to and directly after removal of the sampling material. As it can be seen from **Fig. S4a**, the pre sampling skin resistance values are high and are characterized by substantial variations, most likely caused by inter- and intrapersonal biological variability. Application of sampling materials for 2 h caused a significant decrease in the resistance of the skin barrier as summarized in **Fig. S4b**. This means, that after removal of the sampling material, ion transport across the skin membrane was less restricted[10]. In the case of hydrogels (AGR, CHI and AGC) and hydrated starch (STR), the decrease in skin resistance was due to increased hydration of the skin barrier at the sampling site caused by contact with the sampling material. These results are in a good agreement with our previous study where the dynamics of skin hydration was investigated by means of EIS measurements; almost full skin hydration occurred in 60 min[11]. In case of TPS, the decrease in skin resistance was caused by removal of the most superficial and driest layers of the SC. The results of EIS measurements are summarized in **Table S8**.

The change in skin resistance, shown in **Fig. S4c**, was calculated to compare the influence of the different sampling techniques on the electrical properties of the skin barrier. As it can be seen, application of CHI for 2 h had the strongest influence on the electrical properties of the skin, whereas the effect of TPS was the weakest. The relative change in skin resistance caused by CHI was significantly different compared to the corresponding change caused by other sampling techniques, except for AGC. No significant difference was observed between AGR, STR, and TPS. The resistance of the skin towards flow of ions is strongly dependent on the hydration level of the SC. Obviously, sampling by TPS would not hydrate the skin since adhesive tape does not contain water. The decrease in skin resistance after three tape strips was caused by removal of the outermost and driest layers of the SC, which are known to have high electrical resistance[2,5]. On the other hand, sampling of analytes with hydrogels and STR was performed over 2 h, implying that the difference between materials on their impact on skin resistance could be due to the fact that water content was not the same in these materials (see **Table 1**). An additional factor could be the difference in the ability of materials to release water. CHI loses approximately 69 ± 6 % of its weight during a 2 h skin application, while AGR, AGC or STR lose on average 17 ± 4 %, 13 ± 4 %, and 11 ± 14 %, respectively (see **Table S9**). In conclusion, hydrogels applied on skin for 2 h considerably reduced skin resistance, equalizing this physical barrier property among sampling sites and between individuals. Sampling approaches, which reduce skin resistance variability might be beneficial in reducing variability of collected analytes as fluxes of hydrophilic compounds through skin usually correlate with the reciprocal of skin resistance[12].

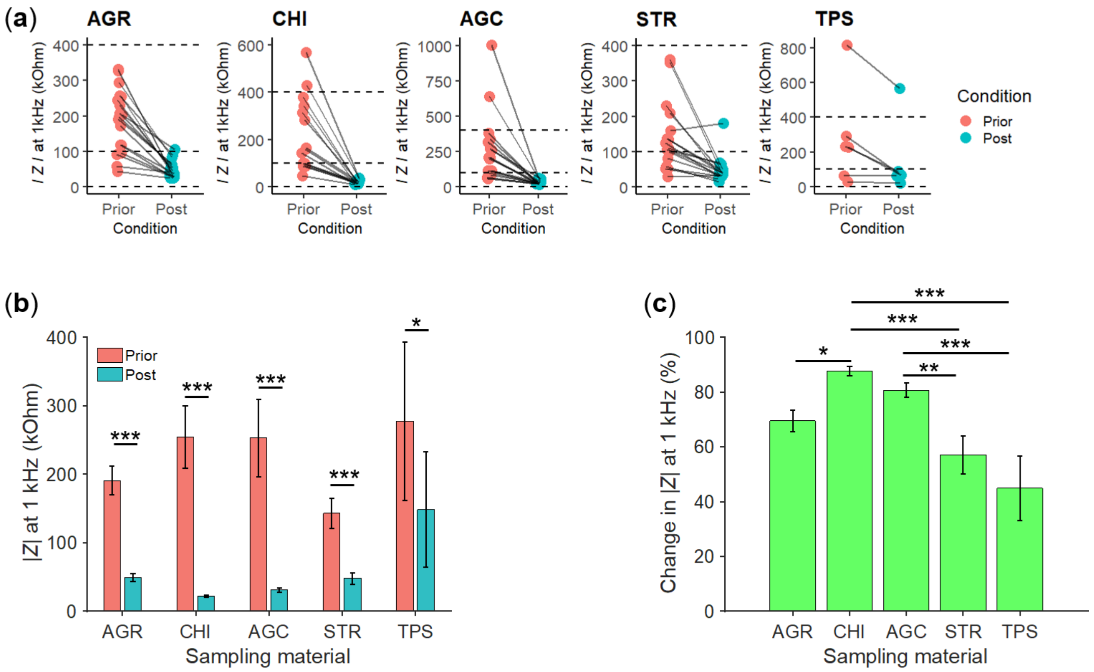

Fig. S4. Skin resistance, evaluated by absolute impedance at 1 kHz (a). The comparison between the mean skin resistance measured prior and post sampling (b). The change in skin resistance caused by different sampling materials (c). Data in (b) and (c) show mean ± SEM (see **Table S8** for the number of observations).

Table S8. The effect of the sampling techniques on the skin resistance evaluated by absolute skin impedance at 1 kHz (kΩ). Measurements were performed prior and post analyte sampling.

**Condition**	**CHI**	**AGR**	**AGC**	**STR**	**TPS**
Prior	254.0 ± 193.2	190.8 ± 87.9	252.9 ± 239.2	142.9 ± 94.1	277.3 ± 283.0
	n = 18	*n* = 18	*n* = 18	*n* = 18	*n* = 6
Post	22.0 ± 7.3	49.4 ± 23.9	31.0 ± 12.2	48.0 ± 35.7	148.4 ± 206.9
	n = 18	*n* = 18	*n* = 18	*n* = 18	*n* = 6
p-value	< 0.001	< 0.001	< 0.001	< 0.001	0.028
Significance	***	***	***	***	*

Table S9. Change of sampling material weight after 2 h topical sampling. The change was calculated by subtracting the weight of the applied material from the weight of the material collected after sampling. The same procedure was followed for sampling at rest (Rest) and while sweating (Sweating).

Sampling material (number of repetitions)	Weight change (%)			
	Rest		Sweating	
	Mean	SD	Mean	SD
AGR (n = 18)	-16.5	4.4	-0.3	11.8
AGC (n =18)	-13.2	3.5	1.6	4.7
STR (n = 18)	-10.6	14.0	13.4	17.0
CHI (n = 3)	-69.0	5.9	-	-

AGR, agarose hydrogel, AGC, agarose and chitosan hydrogel, CHI, chitosan hydrogel, STR, hydrated starch film.

Table S10. CV's (%), coefficient of variation of the analyte quantities and their corresponding ratios estimated in samples collected between individuals, *i.e.*, reflecting inter-individual variability, (a) and in samples collected from the same individuals, *i.e.*, reflecting intra-individual variability, (b).

a)

Analytes	Coefficient of variation (CV (%))					
	AGR	AGC	CHI	STR	TPS	CTN
Trp	60.6	62.3	74.2	61.9	54.5	54.6
Phe	68.4	69.0	83.8	60.1	89.8	64.0
Tyr	94.8	109.0	105.0	81.4	72.8	70.1
Kyn	40.5	-	-	32.8	36.3	-
Tyr/Trp	49.8	58.7	54.3	51.4	51.2	87.5
Phe/Trp	27.0	29.7	19.4	34.4	33.6	33.5
Phe/Tyr	33.3	45.5	61.3	39.2	75.5	69.9
Trp/Kyn	40.9	-	-	49.3	50.5	

b)

Analytes	Coefficient of variation (CV (%))					
	AGR	AGC	CHI	STR	TPS	CTN
Trp	42.2	39.6	46.1	20.2	24.3	37.3
Phe	37.3	39.3	48.7	22.9	31.5	32.0
Tyr	36.4	46.8	50.0	32.1	37.9	54.9
Kyn	13.4	-	-	7.2	15.4	-
Tyr/Trp	17.8	24.4	30.3	15.9	24.6	26.0
Phe/Trp	13.1	9.5	14.5	12.9	23.6	24.2
Phe/Tyr	18.1	24.4	36.8	14.6	23.5	42.0
Trp/Kyn	39.6	-	-	13.4	37.9	-

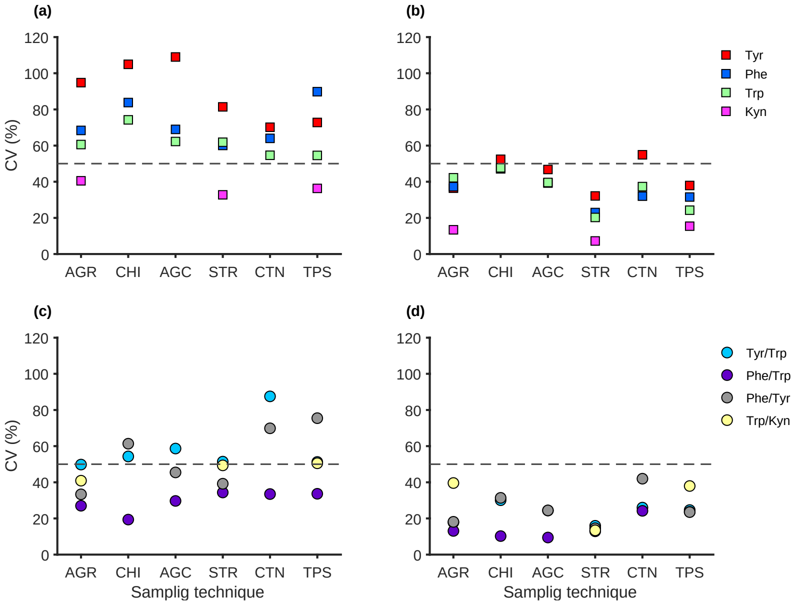

Fig. S5. The variation, evaluated by CV (%), of the absolute quantity of analytes (a, b) and their corresponding ratios (c, d) collected from the skin surface by different sampling techniques. The inter-individual variation is shown in (a, c) and intra-variation in (b, d).

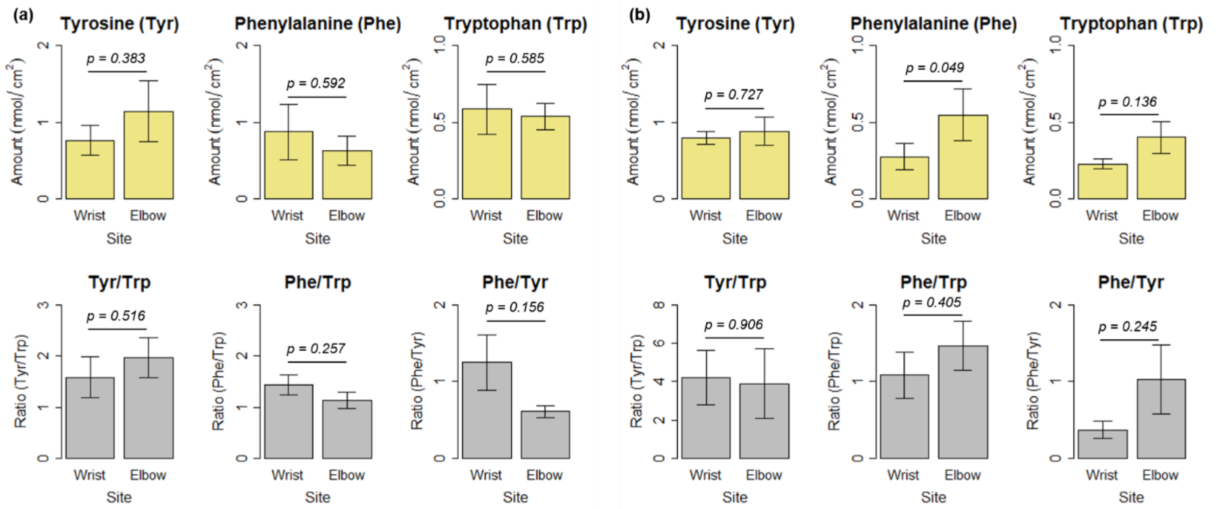

Fig. S6. Comparison between the quantity of analytes and the corresponding ratios determined in tape strip samples collected form the skin surface at a sampling site close to the wrist (*i.e.*, wrist) and close to the elbow (*i.e.*, elbow). Sampling performed at rest (a) and while sweating (b). Data show as mean ± SEM. Comparison between the quantity of analytes (or their corresponding ratios) collected at rest and while sweating was done by performing one-way Anova with Tukey test.

Table S11. Physicochemical characteristics of tryptophan, kynurenine, phenylalanine, and tyrosine. The values of the acid dissociation constant (pKa), molecular weight, and partition coefficients were obtained by using Chemicalize software (ChemAxon) or the PubChem data base.

Permeant	MW (g/mol)	Log P
L-tryptophan 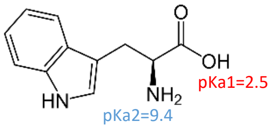	204.23	-1.1[1]
L-kynurenine 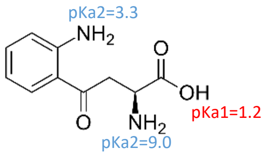	208.21	-1.9[1]
L-phenylalanine 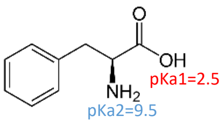	165.19	-1.2[1]
L-tyrosine 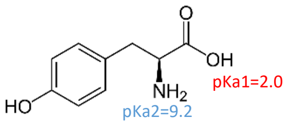	181.19	-2.3[2]

[1]Computed values obtained from Chemicalize software (visited on 2019 August)

[2]Computed value obtained from the PubChem data base (release 2019.06.18)

Table S12. Blood plasma concentration of Tyr, Phe, Trp, and Kyn in six healthy volunteers, three women and three men. Values in the table represent mean ± SD for three technical replicates. The letter W indicates women and the letter M stands for men.

Individual	Concentration (μM)			
	Tyr	Phe	Trp	Kyn
01W	63.9±12.8	45.6±6.1	64.9±0.8	1.5±0.1
02W	72.5±59.2	59.2±5.1	65.9±3.5	1.5±0.2
03W	52.4±8.2	41.0±2.3	47.0±1.8	1.3±0.1
01M	66.6±19.3	42.8±9.4	61.8±11.2	1.6±0.2
02M	69.0±24.6	38.7±7.7	47.6±11.3	1.3±0.2
03M	72.8±23.4	54.0±10.6	63.5±17.6	1.8±0.3

Section S8. Discussion about Tyr, Phe, Trp and Kyn concentration in blood, sweat and skin surface

In order to interpret if biomarkers sampled on the skin surface reflect the systemic concentrations, the comparison between biomarkers levels sampled on the skin surface *vs* blood levels must be performed. Thus, the concentration of Tyr, Phe, Trp, and Kyn was measured in blood plasma of study participants fasting overnight (**Table S12**). On average, the estimated abundance of analytes was as follows: Tyr 66.2 ± 16.5 μM , Phe 46.9 ± 9.8 μM , Trp 58. 5± 11.6 μM, and Kyn 1.5 ± 0.2 μM (mean ± SD, n = 6 for each analyte). Analyte concentrations estimated in our study were in a good agreement with the concentrations reported from a study done on 100 healthy volunteers, where 90.6±22.9 μM of Tyr, 65.2 ± 11.1 μM of Phe, 67.4 ± 10.2 μM of Trp, and 1.8 ± 0.4 μM of Kyn (mean ± SD, n = 100 for each analyte)[13] was measured (**Table S13**). However, there are also a few scientific publications where reported amino acid's concentrations in blood were found to be considerably different from the values estimated in our study. For example, the concentrations of Trp were significantly lower, *i.e*.., 15.5 ± 3.6 μM (mean ± SD, n = 4)[14]. The difference between Trp measurements performed in our study and in the study done by Sylvestre et al (2010), was the preparation of the plasma sample. In our study, prior to LC-MS analysis, proteins were precipitated, whereas in the comparative study[14], proteins were removed by ultrafiltration. Hence, it could be speculated that the difference in Trp concentration between the studies could be attributed to strong Trp binding to plasma proteins which were removed by ultrafiltration[14]. Another example, the noticeably lower reported Tyr concentrations in blood concerned well-trained endurance athletes[15]. Pre-exercise concentration of Tyr was found to be 5.5 ± 3 μM (mean ± SE, n = 10) and post-exercise concentration – 1 ± 1 μM (mean ± SE, n = 10))[15]. It was hypothesized that well trained athletes had lower Tyr levels in general. However results reported from another study done on Olympic athletes indicated that Tyr concentrations in plasma were within the normal range, 53.3 ± 9.6 μM (mean ± SD, n = 52)[16]. Since the age of the participants in both studies were very similar (20-40 years old), this large difference between Tyr concentrations reported by different research groups could be due to differences in analytical protocols including sample handling. These examples emphasize the importance of sample preparation, as well alert about the importance of accuracy and variability of analytical measurements on the analyte quantification. This discussion is important to keep in mind the result interpretation and, especially, for result comparison between different studies.

Table S13. Literature values of Tyr, Phe, Trp, and Kyn measured in blood, skin surface, and sweat. The quantities measured by reverse iontophoresis (RI), in stratum corneum (SC) etc., reported in the literature.

Reference	Sample origin	n	Trp		Kyn		Tyr		Phe		Tyr/Trp		Phe/Trp		Phe/Tyr		Trp/Kyn	
			Mean	SD*	Mean	SD	Mean	SD*	Mean	SD*	Mean	SD	Mean	SD	Mean	SD	Mean	SD
Geisler (2015)[13]	Blood (μM)	58 (M)	69.3	9.9	1.8	0.4	95.0	21.5	67.1	11.8	**1.4**	-	**1.0**	-	**0.7**	-	**38.72**	22.5
	Blood (μM)	42 (W)	64.8	10.2	1.8	0.4	84.5	23.7	62.8	9.49	**1.3**	-	**1.0**	-	**0.7**	-	**36.82**	26.2
Sylvestre (2010)[14]	Blood (μM)	4 (2M+2W)	15.5	3.6	-	-	44.3	11.6	53.3	10.6	**2.9**	-	**3.4**	-	**1.2**	-	-	-
	SC (tape stripping) (nmol/cm²)		8.8	2	-	-	21.5	3	8.5	3.5	**2.4**	-	**1.0**	-	**0.4**	-	-	-
	RI_cathode (nmol/cm²)		3.1	0.6	-	-	7.1	1.5	3.3	1	**2.3**	-	**1.1**	-	**0.5**	-	-	-
	RI_anode (nmol/cm²)		2.3	0.4	-	-	4.5	0.7	2.1	0.7	**2.0**	-	**0.9**	-	**0.5**	-	-	-
	Passive diffusion (nmol/cm²)		2	0.6	-	-	5.4	1.8	2.1	1	**2.7**	-	**1.1**	-	**0.4**	-	-	-
*Dunstan (2016)[15]	Blood (pre-exercise) (μM)	10 (M)	38	3	-	-	4.7	3	45	1	**0.1**	-	**1.2**	-	**9.6**	-	-	-
	Blood (post-exercise) (μM)	10 (M)	31	2	-	-	1.0	1	49	2	**0.03**	-	**1.6**	-	**49**	-	-	-
	Faux sweat (μM)	11 (M)	104	34	-	-	16.0	7.4	157	37	**0.15**	-	**1.5**	-	**9.8**	-	-	-
Mark (2013)[17]	Sweat (armpits) (μM)	6 (M)	7.1	4.8	-	-	30.3	19.1	17.2	11.1	**4.3**	-	**2.4**	-	**0.6**	-	-	-
	Sweat (armpits) (μM)	6 (W)	15.1	5.6	-	-	55.9	20.2	37.8	14.5	**3.7**	-	**2.5**	-	**0.7**	-	-	-
	Blood (μM)	12 (6M+6W)	11.1	6.5	-	-	43.1	23	27.5	16.3	**3.9**	-	**2.5**	-	**0.6**	-	-	-
Bakhshi (2015)[18]	Blood (μM)	60 (M)	58.2	-	-	-	68	-	63.4	-	**1.2**	-	**1.1**	-	**0.9**	-	-	-
	Blood (μM)	60 (W)	51.4	-	-	-	63.2	-	53.2	-	**1.2**	-	**1.0**	-	**0.8**	-	-	-
	SC (Scraped) (μM)	61 (M)	3.2	-	-	-	9.6	-	7.8	-	**3.0**	-	**2.4**	-	**0.8**	-	-	-
	SC (Scraped) (μM)	61 (W)	2.5	-	-	-	6.6	-	4.6	-	**2.7**	-	**1.9**	-	**0.7**	-	-	-
Stoffers (2020)[19]	Skin surface	10	8.8	9.5	-	-	36.3	44.1	22.2	29	**4.1**	-	**2.5**	-	**0.6**	-	-	-

W, woman; M, man. * SE was reported instead of SD.

Section 9. Comparison of the ratios of analytes collected from skin surface at rest, when sweating, and in blood plasma The statistical analysis of the results summarized in **Fig. S7** indicates that there is no statistically significant difference for the majority of amino acid ratios sampled from the skin surface at rest, when sweating, and blood samples (**Table S14**). However, in this study, the statistical analysis of the ratios relied on too few samples collected from skin surfaces or blood. Hence, owing to the limited number of observations, inferences regarding the absence or presence of statistically significant differences may be masked by sampling variations. For example, all ratios between the amino acids estimated in samples collected by TPS at rest and while sweating showed no statistically significant difference compared to blood samples. However, a closer look at the data reveals that owing to high sampling variability no statistical difference was observed, even though the mean values of the amino acid ratios were different between the samples collected from the skin surface by TPS and blood (**Table 2**). Comparison of Tyr/Trp, Phe/Trp, and Phe/Tyr ratios estimated in the samples collected from skin surfaces at rest and skin surfaces while sweating *vs* blood plasma, showed that the ratios, in most cases, were statistically similar with no striking differences (**Fig. S7**, **Table 2**).

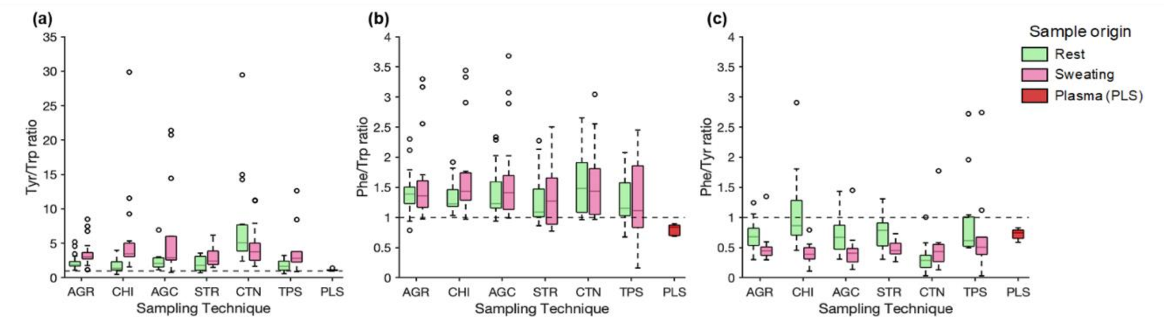

Fig. S7. The ratios of amino acids determined in the samples collected from the skin surface at rest, while excessively sweating, and in blood plasma (PLS). The boundaries of the boxplot represent the first and third quartiles, the thick bar shows the median value, and the vertical line reports the range of the observed values. Data points above or below maxima/minima (vertical line of the boxplot) are considered as potential outliers. The exact measured values can be found in **Table 2**.

Table S14. Comparison between the ratios in blood vs skin surface (*p*-values). Comparison between the ratios of analytes determined in blood plasma and in samples collected from the skin surface by different sampling techniques at rest (a) or while sweating (b). *p*-values obtained by performing two-sample t-test, without and with application of Bonferoni correction for multiple testing (p-value$_{Bonf}$).

(a) Sampling from skin surface at rest

Skin surface at rest				
Tyr/Trp		*p*-value	p-value$_{Bonf}$	
	AGR	0.024	0.145	n.s.
	AGC	0.046	0.275	n.s.
	CHI	0.181	1.087	n.s.
	CTN	0.029	0.175	n.s.
	TPS	0.119	0.713	n.s.
	STR	0.054	0.322	n.s.
Skin surface at rest				
Phe/Trp		*p*-value	p-value$_{Bonf}$	
	AGR	0.001	0.006	**
	AGC	0.002	0.013	*
	CHI	8.9×10^{-5}	0.001	**
	CTN	0.002	0.014	*
	TPS	0.017	0.102	n.s.
	STR	0.015	0.091	n.s.
Skin surface at rest				
Phe/Tyr		*p*-value	p-value$_{Bonf}$	
	AGR	0.726	4.36	n.s.
	AGC	0.878	5.27	n.s.
	CHI	0.726	4.36	n.s.
	CTN	0.726	4.36	n.s.
	TPS	0.451	2.70	n.s.
	STR	0.745	4.47	n.s.

(b) Sampling at skin surface while sweating

Skin surface while sweating				
Tyr/Trp		*p*-value	p-value$_{Bonf}$	
	AGR	0.007	0.040	*
	AGC	0.083	0.496	n.s.
	CHI	0.101	0.607	n.s.
	CTN	0.013	0.079	n.s.
	TPS	0.081	0.485	n.s.
	STR	0.005	0.027	*
Skin surface while sweating				
Phe/Trp		*p*-value	p-value$_{Bonf}$	
	AGR	0.015	0.088	n.s.
	AGC	0.014	0.085	n.s.
	CHI	0.008	0.045	*
	CTN	0.007	0.045	*
	TPS	0.150	0.900	n.s.
	STR	0.024	0.143	n.s.
Skin surface while sweating				
Phe/Tyr		*p*-value	p-value$_{Bonf}$	
	AGR	0.029	0.174	n.s.
	AGC	0.032	0.191	n.s.
	CHI	5.1×10^{-5}	0.000	***
	CTN	0.139	0.833	n.s.
	TPS	0.978	0.279	n.s.
	STR	0.004×10^{-1}	0.303	n.s.

Table S15. The effect of IFN-γ and UV-B radiation treatment of skin model on Tyr/Trp, Phe/Trp, Tyr/Phe, and Trp/Kyn ratios. The effect of IFN-γ and UV-B radiation treatment of a reconstituted human epidermis/dermis model. Tyr/Trp, Phe/Trp, Tyr/Phe, and Tr/Kyn ratios were estimated in the culture medium after the skin model stimulations. The ratios were quantified 48 h after the IFN-γ treatment. UV-20s and UV-40s indicate UV-B irradiation of reconstituted human epidermis for 20 and 40 seconds, respectively. In this case, the ratios were determined 48 h after the UV-B treatment.

Treatment	Tyr/Trp	Phe/Trp	Phe/Tyr	Trp/Kyn	**Change (x/ times) [Compared to Ctrl]**			
					**Tyr/Trp**	**Phe/Trp**	**Tyr/Phe**	**Trp/Kyn**
**Control**	7.9	14.0	1.8	3.9				
**IFN-γ 10 ng/mL**	145	255	1.8	0.04	**↑ 18**	**↑ 18**	**1**	**↓ 98**
**IFN-γ 20 ng/mL**	149	273	1.8	0.05	**↑ 19**	**↑ 19**	**1**	**↓ 77**
**IFN-γ 50 ng/mL**	209	382	1.8	0.04	**↑ 26**	**↑ 27**	**1**	**↓ 99**
**UV-20s** (40 mJ/cm^2)	8.4	14.6	1.8	2.6	**1**	**1**	**1**	**1**
**UV-40s** (80 mJ/cm^2)	7.7	13.2	1.7	3.2	**1**	**1**	**1**	**1**

References

(1) Birgersson, U.; Birgersson, E.; Aberg, P. *Physiol. Meas.* **2011**, *31* (1), 1–19. https://doi.org/10.1088/0967-3334/32/1/001.

(2) Clar, E. J.; Her, C. P.; Sturelle, C. G. *J. Soc. Cosmet. Chem.* **1975**, *26* (7), 337–353.

(3) Yamamoto, T.; Yamamoto, Y. **1976**, *14* (2), 151–158. https://doi.org/10.1007/BF02478741.

(4) Kalia, Y. N.; Pirot, F.; Guy, R. H. *Biophys. J.* **1996**, *71* (5), 2692–2700. https://doi.org/10.1016/S0006-3495(96)79460-2.

(5) Pliquett, U. *Biophys. Chem.* **1996**, *58* (1–2), 205–210. https://doi.org/10.1016/0301-4622(95)00099-2.

(6) Björklund, S.; Ruzgas, T.; Nowacka, A.; Dahi, I.; Topgaard, D.; Sparr, E.; Engblom, J. *Biophys. J.* **2013**, *104* (12), 2639–2650. https://doi.org/10.1016/j.bpj.2013.05.008.

(7) Åberg, P.; Nicander, I.; Hansson, J.; Geladi, P.; Holmgren, U.; Ollmar, S.; Member, S. *IEEE Trans. Biomed. Eng.* **2004**, *51* (12), 2097–2102. https://doi.org/10.1109/TBME.2004.836523.

(8) Morin, M.; Jankovskaja, S.; Ruzgas, T.; Henricson, J.; Anderson, C. D.; Brinte, A.; Engblom, J.; Björklund, S. *Pharmaceutics* **2022**, *14* (2), 313.

(9) Akdeniz, M. D.; Gabriel, S.; Lichterfeld-kottner, A. D.; Kottner, J. D. *Br. J. Dermatol.* **2018**, *179* (5), 1049–1055. https://doi.org/10.1111/bjd.17025.

(10) Björklund, S.; Pham, Q. D.; Jensen, L. B.; Knudsen, N. Ø.; Nielsen, L. D.; Ekelund, K.; Ruzgas, T.; Engblom, J.; Sparr, E. *J. Colloid Interface Sci.* **2016**, *479*, 207–220. https://doi.org/10.1016/j.jcis.2016.06.054.

(11) Morin, M.; Ruzgas, T.; Svedenhag, P.; Anderson, C. D.; Ollmar, S.; Engblom, J.; Björklund, S. *Sci. Rep.* **2020**, *10* (1), 17218. https://doi.org/10.1038/s41598-020-73684-y.

(12) Jankovskaja, S.; Labrousse, A.; Prévaud, L.; Holmqvist, B.; Brinte, A.; Engblom, J.; Rezeli, M.; Marko-Varga, G.; Ruzgas, T. *Microchim. Acta* **2020**, *187* (12), 656. https://doi.org/10.1007/s00604-020-04633-9.

(13) Geisler, S.; Mayersbach, P.; Becker, K.; Schennach, H.; Fuchs, D.; Gostner, J. M. *Pteridines* **2015**, *26* (1), 31–36. https://doi.org/10.1515/pterid-2014-0015.

(14) Sylvestre, J. P.; Bouissou, C. C.; Guy, R. H.; Delgado-Charro, M. B. *Br. J. Dermatol.* **2010**, *163* (3), 458–465. https://doi.org/10.1111/j.1365-2133.2010.09805.x.

(15) Dunstan, R. H.; Sparkes, D. L.; Dascombe, B. J.; Macdonald, M. M.; Evans, C. A.; Stevens, C. J.; Crompton, M. J.; Gottfries, J.; Franks, J.; Murphy, G.; Wood, R.; Roberts, T. K.; Rh, D.; Dl, S. *PLoS One* **2016**, *11* (12), e167844. https://doi.org/10.1371/journal.pone.0167844.

(16) Kingsbury, K. J.; Kay, L.; Hjelm, M. *Br. J. Sports Med.* **1998**, *32* (1), 25–33. https://doi.org/10.1136/bjsm.32.1.25.

(17) Mark, H.; Harding, C. R. *Int. J. Cosmet. Sci.* **2013**, *35* (2), 163–168. https://doi.org/10.1111/ics.12019.

(18) Bakhshi, H.; Sj, H.; Mahmoodi, M.; Mansoori, P. *Int. J. Adv. Res.* **2015**, *3* (1), 643–653.

(19) Stoffers, K. M.; Cronkright, A. A.; Huggins, G. S.; Baleja, J. D. *Anal. Chem.* **2020**, *92* (18), 12467–12472. https://doi.org/10.1021/acs.analchem.0c02274.
